# Stabilization of Condensate Interfaces Using Dynamic Protein Insertion

**DOI:** 10.1021/jacs.5c03740

**Published:** 2025-05-24

**Authors:** Yannick H. A. Leurs, Sanne N. Giezen, Yudong Li, Willem van den Hout, Jay Beeren, Linn J. M. van den Aker, Ilja K. Voets, Jan C. M. van Hest, Luc Brunsveld

**Affiliations:** † Laboratory of Chemical Biology, Department of Biomedical Engineering, 3169 Eindhoven University of Technology, Eindhoven, 5612 AZ, The Netherlands; ‡ Bio-Organic Chemistry, Departments of Biomedical Engineering and of Chemical Engineering and Chemistry, Eindhoven University of Technology, Eindhoven, 5612 AZ, The Netherlands; § Laboratory of Self-Organizing Soft Matter, Department of Chemical Engineering and Chemistry, Eindhoven University of Technology, Eindhoven, 5612 AZ, The Netherlands; ∥ Institute for Complex Molecular Systems (ICMS), Eindhoven University of Technology, Eindhoven, 5612 AZ, The Netherlands

## Abstract

Coacervates have been widely used to mimic membraneless organelles (MLOs). However, coacervates without a membrane or stabilizing surface do not feature the same level of stability as MLOs. This study shows that specifically engineered surface-active proteins can interact with the interface of polypeptide coacervates, conferring resistance to coacervate dissolution and fusion. Modulating the molecular characteristics of these coacervate stabilizing proteins highlighted that their dimerization aids in achieving effective interface stabilizers. Cryo-TEM imaging showed a densely packed protein monolayer at the coacervate-liquid interface, while single-molecule super-resolution microscopy captured the dynamic nature of this protein layer, with the proteins rapidly (un)­docking and moving across the coacervate interface within milliseconds. These findings suggest a dynamic form of coacervate stabilization driven by transient protein interactions at the condensate interface. This unique form of coacervate stabilization not only provides a new approach to developing stable and dynamically exchanging synthetic condensate systems but, as model systems, can also significantly contribute to our understanding of the mechanisms underlying the temporal stability of MLOs in nature.

## Graphical abstract

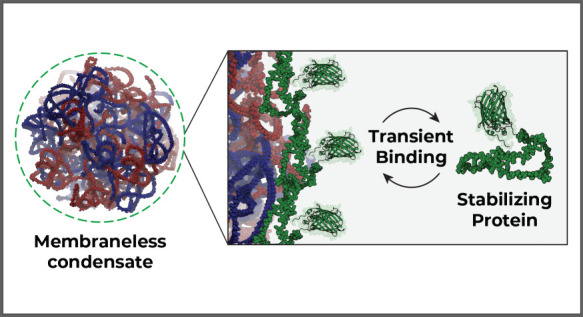

Membraneless organelles (MLOs) are cellular compartments formed via liquid–liquid phase separation (LLPS), driven by multivalent interactions between proteins and/or nucleic acids.^1^ MLOs are crucial for the regulation of many cellular processes, such as the response against stress.^2,3^ Besides the cell biology perspective, also much attention has been paid to the physicochemical basis of MLO formation and stability.^4,5^ However, the dynamic, transient character of these biocondensates remains not fully understood.^6^ In particular, the condensate interface has become a topic of intensive investigation recently to elucidate among others the cause of MLO stability inside living cells.^7–9^

One approach to gain more insight into this behavior is to study synthetic complex coacervates as models for MLOs.^10–12^ Complex coacervates provide a versatile bottom-up platform to recreate MLO-like systems with similar and tunable physiochemical properties.^13^ They have been employed to investigate macromolecule accumulation,^14^ enzymatic regulation of protein interactions,^15^ and enhanced enzymatic activity within condensates.^16–18^ Moreover, multiphasic coacervates containing various substructures have further replicated internal MLO architecture and organization.^19–21^

Two categories of coacervates exist: membraneless systems, which reflect the dynamic, transient nature of some MLOs^22–24^ but typically lack long-term stability,^13,25^ and membrane-functionalized or cross-linked systems, which offer enhanced stability and enable the design of intricate and controllable structures.^26,27^ Various strategies to impart membrane-functionality have been developed, including the use of lipids,^28,29^ polymers,^30^ surfactants,^31^ protein–polymer conjugates,^32^ protein cages,^33^ and inorganic nanoparticles.^34^ However, such membranes reduce coacervate dynamicity, limiting their resemblance to natural condensates.^13^

Here, we present a novel protein-based concept to dynamically stabilize polypeptide complex coacervates. In contrast to static encapsulation or synthetic surfactants, our approach uses dynamic coacervate stabilizing proteins that form reversible, mobile interfacial layers. Fusion proteins featuring a charged intrinsically disordered protein domain and a well-folded buoyant protein domain transiently interact with the coacervate interface, providing stability against coacervate dissolution and coalescence while retaining dynamicity. This approach therefore combines the inherent dynamicity of membraneless coacervates with enhanced robustness, which makes it an interesting platform for MLO research. It also demonstrates the versatility of the condensate structure, which has unique interfacial properties that allow its stabilization via alternative approaches than classical membrane formation by amphiphiles.

Over the past years we have built up much experience in the construction of complex coacervates using modified amylose derivatives.^30^ In order to create an effective MLO mimic in our lab, complex coacervates composed solely of polypeptides were systematically screened. The combination of poly­(l-lysine) and poly­(l-aspartic acid) proved to be the most robust, as these polypeptides could be combined efficiently over a wide range of polymer chain lengths (Supplementary Figures S1 and S2).^35,36^ However, although condensate formation was observed, the droplets lacked stability, coalesced, or wetted the substrate. To achieve stability and to complement the sole protein-based character of these condensates, a fusion protein was designed to act as an amphiphile, in which a positively charged disordered peptide was combined with a (slightly) negatively charged stably folded protein. The flexible cationic sequence should interact with the negatively charged condensate surface, with the folded protein domain preventing internalization.^37,38^ For the cationic part, we chose an intrinsically disordered protein (IDP) sequence from Tau, known for stabilizing cellular structures via its positively charged microtubule-binding region.^39^ Tau was fused to Green Fluorescent Protein (GFP), to provide, besides steric and electrostatic repulsion with the condensate surface, also easy visualization of the fusion protein (Figure 1A).

**Figure 1:**
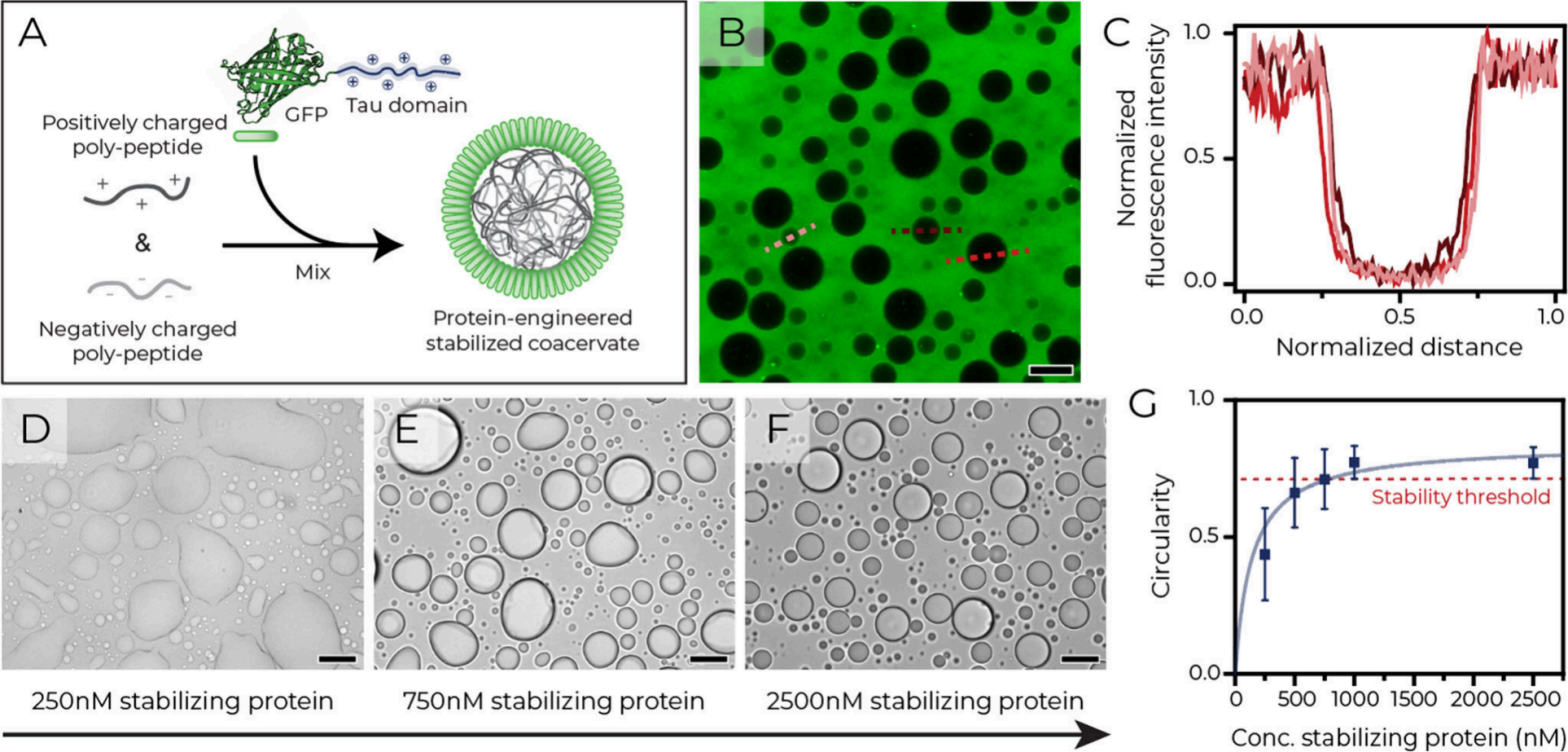
Protein-stabilized polypeptide coacervates. (A) Illustration of GFP-Tau stabilizing phase-separated polypeptides. (B) Confocal micrograph of GFP-Tau in the dilute phase and (C) corresponding fluorescence intensity along the dotted lines. (D–F) Brightfield micrographs showing morphology changes with increasing GFP-Tau concentrations. (G) Circularity as a function of GFP-Tau concentration (mean ± SD, *n* ≥ 50). Scale bars: 30 μm.

The resulting GFP-Tau fusion was tested for stabilizing poly­(l-lysine)_100_/poly­(l-aspartic acid)_250_ coacervates, prepared with excess poly­(l-aspartic acid) to complement the overall positive charge of GFP-Tau. By mixing GFP-Tau with the polypeptides before the onset of phase separation, coalescence and Ostwald ripening were drastically reduced so that stable droplets were formed that remained intact at room temperature for over a week (Figure 1A,B). Droplets in the absence of GFP-Tau rapidly dissolved, wetted, or fused within minutes (Supplementary Figure S3). Remarkably, confocal microscopy showed GFP-Tau to be distributed throughout the dilute phase rather than exclusively at the interface, suggesting a mechanism distinct from classical surfactants (Figure 1B,C). To further probe this stabilizing mechanism, we examined GFP-Tau interactions with negatively charged, neutral, and positively charged coacervates. Negatively charged coacervates yielded stable droplets, while neutral ones showed partial wetting, and positively charged coacervates exhibited increased instability and wetting along with reduced droplet number and size (Supplementary Figure S4A–D). Moreover, using a Tobacco Etch Virus (TEV) protease site engineered between GFP and Tau, we found that cleavage of the covalent linkage abolished droplet stabilization, indicating that the disordered and folded domains must remain tethered (Supplementary Figure S4E–G).

The stability of the coacervates was tunable by the GFP-Tau concentration (Figure 1D–F). To more quantitatively analyze droplet stability, we employed the circularity parameter; a common metric for coacervate morphology (Methods, eq 1).^40,41^ Confocal microscopy of complex coacervates also featuring a small amount of Cy5-labeled poly­(l-lysine)_100_ (Supplementary Figure S5) revealed that circularity increased with GFP-Tau concentration (Figure 1G). A stability threshold (red line, Figure 1G, Methods, eq 2) indicated that approximately 1 μM GFP-Tau was required for robust stabilization of 50 μM total polypeptides. Finally, to demonstrate the generality of our approach, we fused a negatively charged region of α-synuclein (aSyn) to a positively engineered GFP (GFP-aSyn), which effectively stabilized net-positively charged coacervates (Supplementary Figure S6).

GFPs are known to feature dimerization tendencies and we hypothesized that such valency enhancement may contribute to its buoyancy efficiency in GFP-Tau. To evaluate this hypothesis, fusion proteins with distinct dimerization properties were designed (Figure 2A). The strongly dimerizing Glutathione S-Transferase (GST, PDB: 1PKW)^42^ was chosen to produce GST-Tau as a model for strong dimerization, while monomeric ultrastable GFP (muGFP, PDB: 5JZL)^43^ was used as part of the nondimerizing variant muGFP-Tau. Both constructs retained GFP’s overall charge and size, ensuring that any differences arose primarily from dimerization behavior.

**Figure 2:**
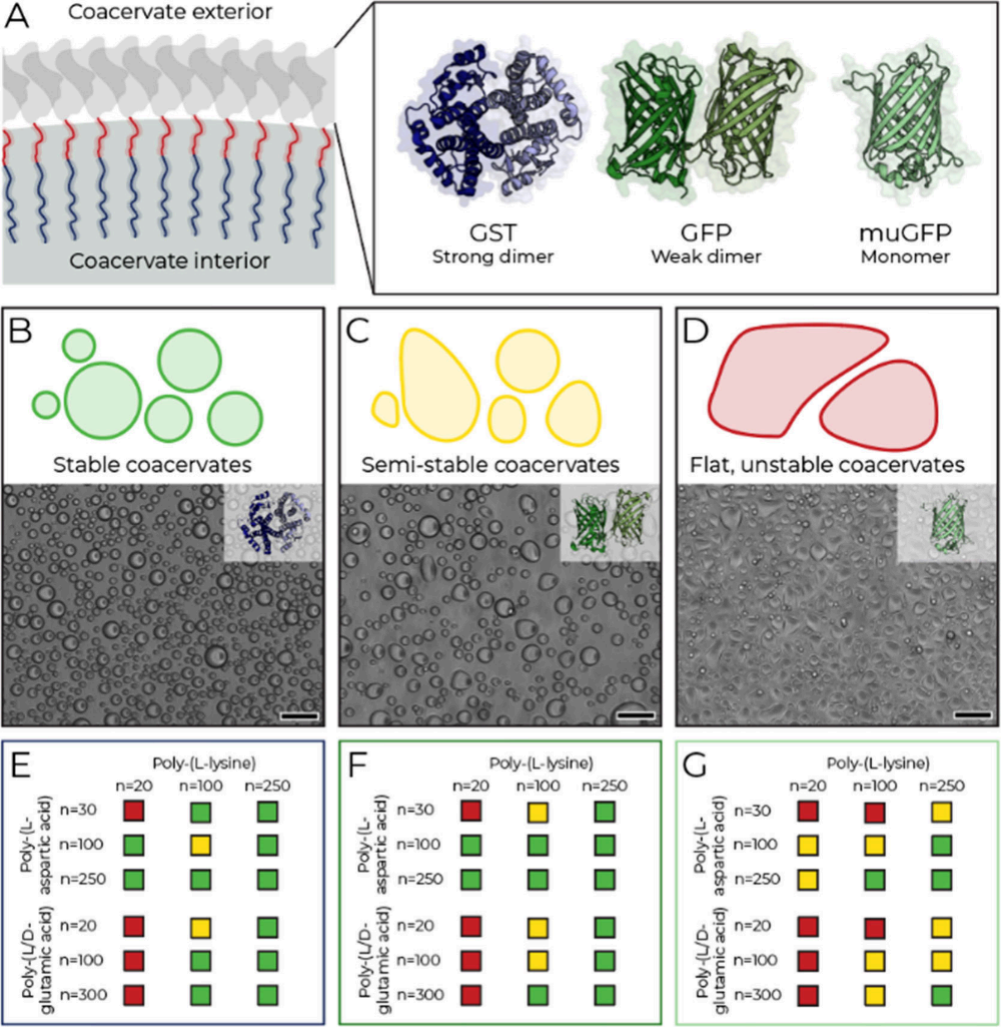
Stabilizer dimerization enhances coacervate stability. (A) Dimerization is tested by substituting GFP (PDB: 5NHN) in GFP-Tau with dimerizing GST (PDB: 1PKW) or nondimerizing muGFP (PDB: 5JZL). (B–D) Brightfield micrographs of poly­(l-aspartic acid)_30_/poly­(l-lysine)_100_ coacervates with GST-Tau (B), GFP-Tau (C), or muGFP-Tau (D). (E–G) Classification of stabilization effects of GST-Tau (E), GFP-Tau (F), and muGFP-Tau (G) across 18 polypeptide combinations (green, stable; yellow, semistable; red, unstable). Scale bars: 50 μm.

We examined coacervates formed from poly­(l-aspartic acid)_30_ and poly­(l-lysine)_100_ in the presence of each fusion protein (Figure 2B–D). As with previous experiments, these coacervates were prepared with excess negative charge. Analyses of circularity and wetting behavior revealed that GST-Tau (Figure 2B) stabilized this composition most effectively; GFP-Tau (Figure 2C) was moderately effective, whereas muGFP-Tau (Figure 2D) failed to stabilize.

Next, the set of fusion proteins was screened against a library of phase-separating polypeptide combinations with varying chain lengths (Figure 2E–G). Coacervates were classified by wetting behavior and circularity as stable (green), semistable (yellow), or unstable (red), using a deliberately conservative scheme in which any deviation from fully stable behavior was categorized as semistable. Only a few coacervate compositions were stabilized by muGFP-Tau, whereas both GFP-Tau and GST-Tau stabilized a wide range of compositions. Notably, even within the same semistable classification, muGFP-Tau showed more pronounced droplet wetting and instability compared to other stabilizing proteins (Supplementary Figures S7–S9). Similar effects were observed when buoyancy protein dimerization was suppressed using supercharged GFP constructs (+15GFP-Tau or −25GFP-Tau; Supplementary Figures S10–12). It should be noted that GFP dimerizes in the high micromolar range,^44^ whereas GST forms dimers in the low nanomolar range.^42^ At the 2.5 μM stabilizer concentration used, GST-Tau likely exists predominantly in the dimeric state in both the bulk phase and at the coacervate interface. In contrast, GFP-Tau is likely to mainly dimerize at the coacervate surface. This could explain the effective coacervate stabilization by GFP-Tau. The efficiency of coacervate stabilization by dynamic protein insertion is thus enhanced by dimerizing buoyancy groups, potentially by improving interface packing, enabling bivalent anchoring, and increasing hydrodynamic radius. Consistent with this, GST-Tau supported week-long morphological and dynamic stability in synthetic polypeptide coacervates (Supplementary Figures S13–S14), and also stabilized compositionally distinct, biologically derived coacervates formed with the Nephrin intracellular domain (NICD) (Supplementary Figure S15)

The insights into the molecular features that determine the stabilizing ability of the fusion proteins prompted the study of their interfacial architecture. We therefore employed cryo-Transmission Electron Microscopy (cryo-TEM) to study the structural organization of the stabilizing proteins at the coacervate interface. Nonstabilized coacervates showed poorly defined interfaces (Figure 3A-B). In contrast, GST-Tau and GFP-Tau formed well-defined protein membranes at the interface between the dense and dilute phases (Figure 3C–F), with membrane widths of 3.4 ± 0.7 nm and 4.6 ± 0.8 nm, respectively (Figure 3I). These measurements align with the diameters of GFP and GST (Supplementary Figure S16) and support the presence of a protein monolayer at the coacervate interface. As an additional control, a triple-domain fusion protein (mEOS3.2-GST-Tau) with an additional N-terminal buoyancy group (mEOS3.2) formed a thicker monolayer membrane (5.7 ± 0.8 nm), again consistent with a protein monolayer.

**Figure 3:**
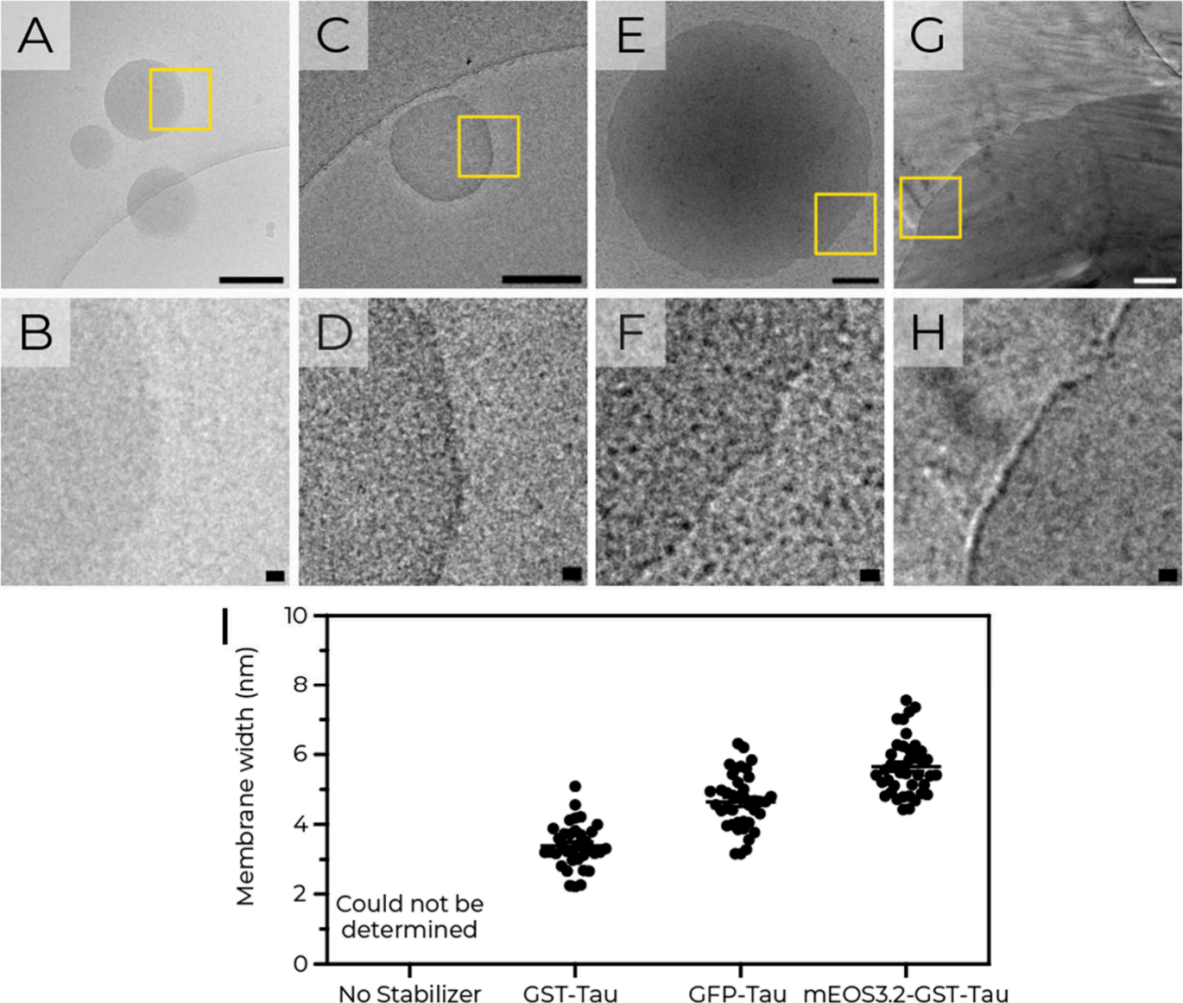
Protein stabilizers form monolayer membranes. (A-B) Representative cryo-TEM micrographs of poly­(l-lysine)_100_/poly­(l-aspartic acid)_250_ coacervates without stabilizers, and with (C–D) GST-Tau, (E-F) GFP-Tau, or (G-H) mEOS3.2-GST-Tau. Lower panels show magnified regions (yellow boxes). (I) Membrane width measurements derived from micrographs (A-H, *n* > 30 measurements). Scale bars: 200 nm (upper), 10 nm (lower).

The dynamics of the protein monolayer at the coacervate interface were probed using single-particle tracking photoactivated localization microscopy (sptPALM) under highly inclined and laminated optical sheet (HILO) illumination.^45^ This method enabled high-resolution imaging of individual proteins and bulk populations by switching their fluorescence.^46^ For this purpose, the stabilizing protein mEOS3.2-GST-Tau was used, with its mEOS3.2 domain capable of photoconverting from green (516 nm) to red (581 nm) under UV irradiation (390 nm) (Figure 4A). To reduce fluorophore density, mEOS3.2-GST-Tau was mixed with unlabeled GST-Tau. Its accumulation at the coacervate interface was monitored in the green channel (Figure 4B), while photoconverted mEOS3.2-GST-Tau enabled high-resolution visualization of the membrane in the red channel (Figure 4C).

**Figure 4:**
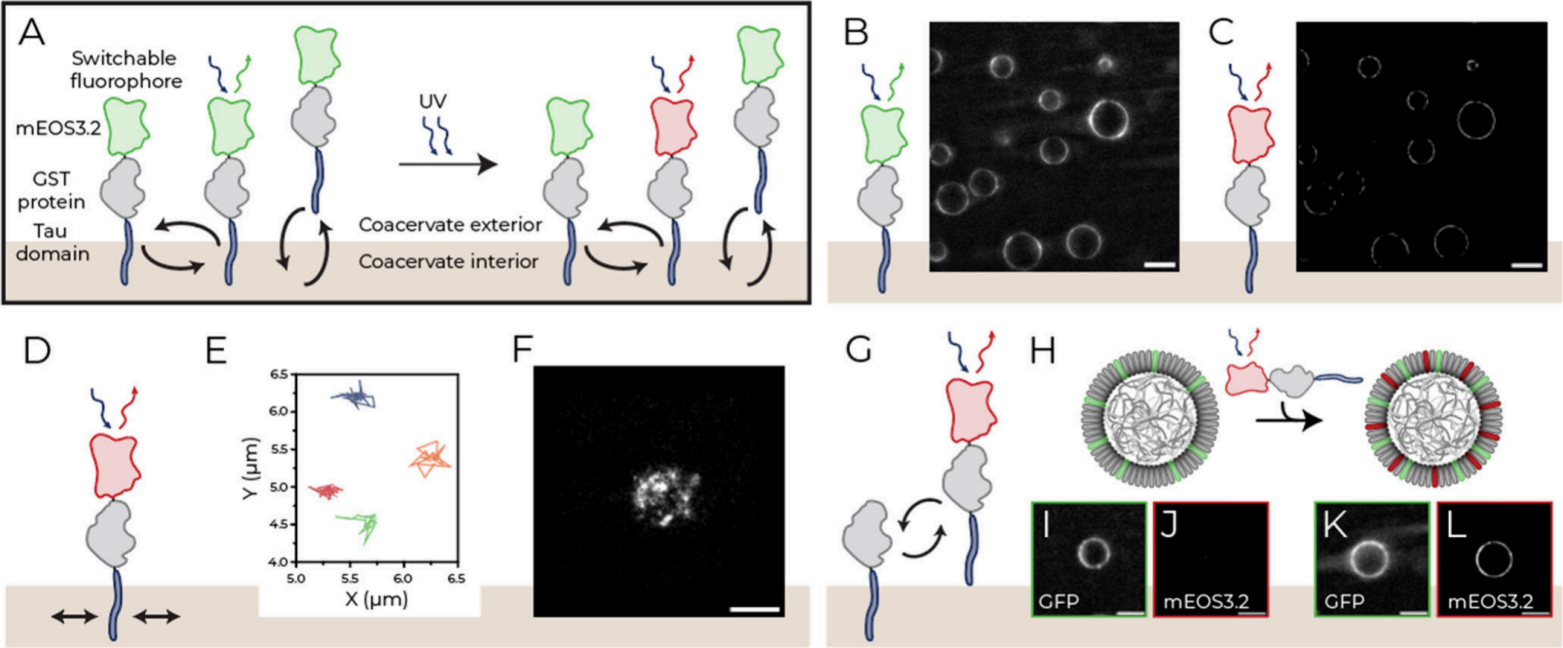
Dynamic behavior of stabilizing proteins at the coacervate interface. (A) Proteins interact via diffusion, docking, and undocking. Photoconvertible mEOS3.2-GST-Tau (green-to-red) enables (B) bulk and (C) single-molecule imaging. (D) Diffusion schematic with (E) tracks and (F) reconstruction. (G) Protein exchange schematic and (H) experimental setup: GST-Tau/GFP-Tau coacervates imaged (I-J) before and (K-L) after mEOS3.2-GST-Tau addition, confirming interfacial protein exchange. Scalebars: 10 μm (B–C), 2 μm (F), 1 μm (I-L).

To analyze the dynamics of surface-bound stabilizers, proteins at the top of the coacervate were traced in the X/Y plane (Figure 4D-F). Imaging at the top provided an X/Y plane area of approximately 1.5 μm^2^ in which 2D diffusion of the proteins could be tracked without going out of focus in the *z*-direction. During acquisition, dynamic movement across the coacervate interface was observed (Supplementary Video V1). In addition to lateral diffusion in the membrane, dynamic incorporation of stabilizing proteins from the bulk solution into the coacervate interface was observed (Figure 4G,H), as the addition of photoconvertible mEOS-GST-Tau caused an increase in the red channel fluorescence over a time scale of minutes (Figure 4I–L). GST-Tau-stabilized coacervates also remain permeable to externally added cargo (Supplementary Figure S17).

We have demonstrated that fusion proteins with an intrinsically disordered and charged domain with a folded buoyant headgroup can selectively interact with artificial polypeptide-based coacervates, providing stability against droplet coalescence. Our findings reveal key design principles for effective surface-active proteins: (1) a disordered region with net charge opposite to the coacervate, promoting electrostatic binding; (2) a stably folded globular domain that is excluded from the coacervate interior by steric and electrostatic effects; and (3) moderate dimerization or multivalency to enhance interfacial retention. At the coacervate interface, the stabilizing proteins form a monolayer that remains remarkably dynamic, with proteins continuously (un)­docking and migrating across the surface. This dynamicity raises intriguing questions about whether similar mechanisms occur in cellular contexts, where specific cytoplasmic proteins with similar characteristics might regulate MLO stability.

The protein membrane concept presented here provides a robust and modular foundation for developing dynamic artificial cell models. By tuning stabilizer properties, coacervate systems can be designed with customized charge, density, and fluidity. Combined with the dynamicity of the coacervate interface, and thus overcoming concurrent challenges with less dynamic and apolar membranes, comprehensive studies can be performed to understand how macromolecules are partitioned into condensates.

## Supplementary Material





## Notes

Materials and methods, supplementary figures (phase separating behavior, brightfield images, electrostatic interactions, circularity determination, protein engineering, dimerization suppression, fluorescence images, micrographs), protein constructs and properties, supplementary references (PDF)
Protein dynamics at the interface (MP4)

## Abbreviations

MLO: membraneless organelle
LLPS: liquid–liquid phase separation
IDP: intrinsically disordered protein
GFP: green fluorescent protein
TEV: tobacco etch virus
GST: glutathione S-transferase
muGFP: monomeric ultrastable green fluorescent protein
Cryo-TEM: cryogenic transmission electron microscopy
sptPALM: single-particle tracking photoactivated localization microscopy
HILO: highly inclined and laminated optical sheet
