# Supplementary material for: Stabilization of Condensate Interfaces Using Dynamic Protein Insertion
Source: J Am Chem Soc. 2025 May 24;147(22):18412–8. doi: 10.1021/jacs.5c03740 (PMC12147158; doi:10.1021/jacs.5c03740)
Supplement: Supplementary file 1 [file ja5c03740_si_001.pdf]

Supplementary Information

Stabilization of Condensate Interfaces Using Dynamic Protein Insertion

Yannick H.A. Leurs[1,2,4], Sanne N. Giezen[3,4], Yudong Li[2,4], Willem van den Hout[1,2,4], Jay Beeren[1,2], Linn J.M. van den Aker[1,2,4], Ilja K. Voets[3,4], Jan C. M. van Hest*[2,4], Luc Brunsveld*[1,4]

[1] Laboratory of Chemical Biology, Department of Biomedical Engineering, Eindhoven University of Technology, Eindhoven, 5612 AZ, The Netherlands

[2] Bio-Organic Chemistry, Departments of Biomedical Engineering and of Chemical Engineering and Chemistry, Eindhoven University of Technology, Eindhoven, 5612 AZ, The Netherlands

[3] Laboratory of Self-Organizing Soft Matter, Department of Chemical Engineering and Chemistry, Eindhoven University of Technology, Eindhoven, 5612 AZ, The Netherlands

[4] Institute for Complex Molecular Systems (ICMS), Eindhoven University of Technology, Eindhoven, 5612 AZ, The Netherlands

* j.c.m.v.hest@tue.nl, l.brunsveld@tue.nl

Keywords: Coacervates, Condensate Stability, Interface, Biomolecular Condensates, Membraneless Organelles

Contents

1. MATERIALS & METHODS
    a. DNA molecular biology and cloning
    b. Protein expression and purification
    c. TEV cleavage
    d. Coacervate preparation and imaging
    e. Imaging plate surface passivation through BSA coating
    f. FRAP (Fluorescence Recovery After Photobleaching)
    g. Circularity determination
    h. Cryo-TEM imaging
    i. Super-resolution microscopy
2. SUPPLEMENTARY FIGURES
    a. Supplementary Figure S1: Phase separating behavior of various polyelectrolytes, as determined by brightfield microscopy.
    b. Supplementary Figure S2: Representative brightfield images of poly-(L-lysine)/poly-(L-aspartic acid) coacervates imaged on BSA-coated glass slides to minimize substrate-induced wetting and preserve droplet morphology.
    c. Supplementary Figure S3: Brightfield micrographs of coacervates formed from poly-(L-lysine)$_{100}$ and poly-(L-aspartic acid)$_{250}$ in the absence of protein stabilizer.
    d. Supplementary Figure S4: Electrostatic interactions and buoyancy drive coacervate stabilization.
    e. Supplementary Figure S5: Circularity determination from confocal micrographs.
    f. Supplementary Figure S6: Protein engineering of a coacervate stabilizer for positively charged coacervates.
    g. Supplementary Figure S7: The stabilizing effect of 2.5μM GST-Tau against a library of phase-separating polypeptides.
    h. Supplementary Figure S8: The stabilizing effect of 2.5μM GFP-Tau against a library of phase-separating polypeptides.
    i. Supplementary Figure S9: The stabilizing effect of 2.5μM muGFP-Tau against a library of phase-separating polypeptides.
    j. Supplementary Figure S10: Suppressing dimerization through supercharging the buoyancy group reduces coacervate stability.
    k. Supplementary Figure S11: The stabilizing effect of 2.5μM +15GFP-Tau against a library of phase-separating polypeptides.
    l. Supplementary Figure S12: The stabilizing effect of 2.5μM -25GFP-Tau against a library of phase-separating polypeptides.
    m. Supplementary Figure S13: Protein-stabilized coacervates remain morphologically stable over extended time periods.
    n. Supplementary Figure S14: The internal molecular dynamics of GST-Tau-stabilized coacervates remains unchanged over time.
    o. Supplementary Figure S15: Stabilization of NICD-based coacervates using the GST-Tau fusion protein.
    p. Supplementary Figure S16: Size comparison of Green Fluorescent Protein (GFP) and Glutathione S-transferase (GST) based on their crystal structures.
    q. Supplementary Figure S17: Surface-stabilized coacervates remain permeable and support post-formation uptake of cargo molecules.
3. PROTEIN CONSTRUCTS AND PROPERTIES
    a. Supplementary Table 1: Sequences and parameters of the proteins used in this study.
4. SUPPLEMENTARY REFERENCES

1. MATERIALS & METHODS

DNA molecular biology and cloning

The sequence for GFP was derived from UniProt ID P42212 (Green fluorescent protein), the sequence for GST was derived from UniProt ID P08515 (Glutathione S-transferase class-mu 26 kDa isozyme), and the sequence for NICD was derived from UniProt ID O60500 (NPHN_HUMAN). The sequence of muGFP was based on work by Scott et al. (2018).[1] The sequences of -25GFP[2] and +15GFP[3] were obtained from work of the research group of Liu. The sequence for +36GFP matches that reported by Altenburg et al.[4] The sequence for mEOS3.2 was derived from work by Zang et al. (2012).[5] Protein sequences and physiochemical properties are provided in Supplementary Information, Section 3. All DNA was ordered through Genscript or Tsingke in a pET28a vector using NcoI and XhoI restriction sites. Sequences were codon optimized for Escherichia Coli (*E.coli*). The plasmid encoding +15GFP-Tau was cloned in-house using Gibson assembly, as described by the protocols provided by New England Biolabs (NEB, High-Fidelity PCR Master Mix with HF Buffer). If applicable, constructs were verified by Sanger sequencing (Genewiz).

Protein expression and purification

Plasmids were transformed into BL21 (DE3) competent cells (Novagen) using heat shock treatment and cultured in LB medium (50mg/ml kanamycin) at 37°C and 160rpm until an OD of 0.6-1.0. Protein expression was induced with 0.4 mM isopropyl-β-d-thiogalactopyranoside (IPTG), and cultures were incubated overnight at 21˚C and 160rpm. Cells were harvested by centrifugation (8600 rpm, 20 minutes, 4˚C) and resuspended in lysis buffer (100mM Tris, pH 8, 150mM NaCl, 1mM EDTA, 0.5mM TCEP) containing cOmplete™ EDTA-free Protease Inhibitor Cocktail tablets (1 tablet/100 ml lysate) and benzonase (5ml/100ml lysate). Lysis was achieved via sonication (Q500, Qsonica), and cell lysates were cleared by centrifugation (20,000 rpm, 30 minutes, 4˚C). Proteins were purified using Ni-NTA and/or Strep-Tactin affinity chromatography depending on tag configuration. For His-tagged proteins, clarified lysates were applied to Ni-NTA cartridges pre-equilibrated in wash buffer (50 mM HEPES, pH 7.4, 300–600 mM NaCl, 25 mM imidazole, 1 mM DTT), followed by elution with 250–500 mM imidazole. Buffer composition was adjusted as needed; for example, +36GFP was purified using buffers containing 600 mM NaCl to improve solubility. Strep-tagged proteins were purified on Strep-Tactin XT cartridges, eluted with 100 mM biotin in Tris-based buffer, and dialyzed using 10 kDa MWCO membranes (Millipore) into storage buffer (10 mM HEPES, pH 7.4, 150 mM NaCl; 600mM NaCl for +36GFP). For constructs containing an N-terminal His-SUMO fusion, SUMO cleavage was performed during overnight dialysis into storage buffer in the presence of 1:10 (w/w) SUMO hydrolase. Cleaved His-SUMO and undigested fusion proteins were removed by reverse Ni-NTA purification, with the untagged protein collected from the flowthrough. Final proteins were aliquoted, flash-frozen in liquid nitrogen, and stored at –80°C. Protein purity was validated by SDS-PAGE and Q-Tof LC/MS.

TEV cleavage

For TEV cleavage, 1 mg of TEV protease was used per 100 mg of purified protein (GFP-Tau, with an internal TEV cleavage site) in the presence of 1mM of Dithiothreitol (DTT). After overnight incubation at 4°C, the resulting protein mixture contained His-tagged TEV protease, Strep-tagged Tau protein, and tag-free GFP. To separate all cleaved components, a Ni-NTA cartridge (Cytiva) was placed in tandem with a Strep-Tactin XT cartridge, and both were equilibrated with NTA-Wash buffer (50mM HEPES, 300mM NaCl, 25mM Imidazole, pH 7.4). The cleaved protein mixture was looped over the column, whereafter the flowthrough containing tag-free GFP was collected. Afterward, the Ni-NTA cartridge was detached from the Strep-Tactin XT cartridge. The Strep-Tactin XT cartridge was then washed with Strep-Wash buffer (100mM Tris, pH 8, 150mM NaCl, 1mM EDTA) and eluted with Strep-Elution buffer (100mM Tris, pH 8, 150mM NaCl, 1mM EDTA, and 100mM Biotin) to obtain strep-tagged Tau. TEV protease was eluted from the Ni-NTA cartridge using NTA elution buffer (50mM HEPES, 300mM NaCl, 250mM imidazole, pH 7.4). Tag-free GFP and strep-tagged Tau were extensively dialyzed using a 10 kDa molecular weight cut-off (MWCO) membrane (Millipore) to storage buffer (10 mM HEPES pH7.4, 150 mM NaCl), and aliquots were flash-frozen for storage at -80°C. Protein purity was analyzed via SDS-PAGE and Q-Tof LC/MS.

Coacervate preparation and imaging

Poly-(L-lysine)$_{20/100/250}$, poly-(L-aspartic acid)$_{30/100}$, and poly-(L/D-glutamic acid)$_{20/100/300}$ were ordered from Alamanda Polymers. Poly-(L-aspartic acid)$_{250}$ was sourced from Sigma Aldrich. All polymers were dissolved at 25 mg/mL in MilliQ, and aliquots were stored at -30C. Working solutions (20 mM monomer charge concentrations) were stored at 4 °C. For a typical experiment, MilliQ, 1 M HEPES pH 7.4, and 4M NaCl were mixed with the positively charged polymer and stabilizing protein in a 1.5mL tube. The mixture was transferred to a MixMate (Eppendorf) and shaken at 1500 rpm for 6 minutes. While shaking, coacervation was induced after 1 minute by adding the negatively charged polymer. For cargo uptake experiments, cargo was added

either during shaking—30 seconds after addition of the negatively charged polymer—or later, directly in the imaging plate. The final coacervate conditions for all experiments (unless detailed otherwise) was 50mM HEPES, pH 7.4, 100mM NaCl, 2.5 µM stabilizing protein, 3.2mM of positive monomer charge, and 4.8mM negative monomer charge. In experiments with alternatively charged coacervates, the charge ratios of positively and negatively charged polymers varied while maintaining 8mM of total charges across all samples. For coacervates involving the NICD protein, a total charge concentration 1mM was used. The charge ratios for negative, neutral, and positive coacervates were 2:3, 1:1, and 3:2, respectively (positive:negative). For confocal experiments, confocal laser scanning microscopy (Leica TCS SP8) was performed with an HC PL APO CS2 63× water immersion objective (NA 1.20). For confocal microscopy, 100 µL of each sample was loaded on a µ-slide 18-well glass bottom (Ibidi). The system was equipped with a 488 nm laser (used for GFP) or 638 nm laser (used for Sulfo-Cy5) and a hybrid detector (HyD). The laser power and detector gain were optimized to use the maximum number of gray values of the detector. The pinhole was set to 1 Airy Unit for the wavelength of maximum emission for each fluorophore. For brightfield microscopy screening experiments, 100 µL of each sample was loaded on Falcon 96-well clear flat bottom microplates and brightfield images were acquired using a Zeiss Axio Observer D1 microscope coupled with an AxioCamMR3 camera, at an objective with 20× magnification.

All imaging was performed on standardized surfaces: Ibidi µ-slide 18-well glass-bottom slides were used for confocal microscopy and sptPALM experiments, and plastic Falcon 96-well flat-bottom microplates were used for brightfield screening experiments. The only exception was Supplementary Figure S2, where BSA-coated glass slides were used to minimize substrate-induced wetting. Importantly, condensate stabilization by engineered proteins was consistently observed across all tested substrates, whereas no stable formulations were identified in their absence, regardless of surface properties.

Imaging plate surface passivation through BSA coating

For BSA coating, black 96-well glass-bottom microscopy plates (Cellvis, 1.5, P96-1.5H-N) were used and the glass surface was passivated to reduce wetting of the coacervates. To prepare the coating, bovine serum albumin (BSA) was dissolved in MQ at 30 mg/mL and then sterile-filtered through a 0.2 µm filter. A volume of 100 µL of this BSA solution was added to each well. The plates were placed on a MixMate shaker (Eppendorf) and incubated at 500 rpm for 60 minutes at room temperature. After incubation, the BSA solution was discarded, and each well was rinsed three times with 100 µL of MQ water. The plates were then dried overnight, covered with a Kimwipe, and stored at room temperature under a protective cover until use.

FRAP (Fluorescence Recovery After Photobleaching)

FRAP experiments were carried out on coacervate droplets containing 100 nM Cy5-labeled poly-lysine and 250 nM of a supercharged Green Fluorescent Protein (+36GFP). Imaging was performed on a Leica SP8 confocal microscope using a HC PL APO CS2 63× water immersion objective (NA 1.20) at 5× zoom. Photobleaching was performed using the LAS X software interface with PMT detector. Each FRAP acquisition began with 10 pre-bleach images (1024 × 1024 resolution, 600 Hz scan speed, 0.86 s per frame) captured at low laser intensity. A circular region of interest (ROI, 4 µm diameter) was then bleached for 10 iterations using 100% laser power. Recovery was monitored immediately afterward for 400 frames using identical imaging parameters. Image analysis was performed using Python. For each acquisition, intensities were extracted from three fixed-diameter circular ROIs: the bleached region, a reference area within the same droplet, and a background region. The time-dependent intensity of the bleached area was corrected by subtracting background and normalized to the reference intensity over time, following the approach described by Poudyal et al.[6] Recovery curves were fitted using a single-exponential model, from which the recovery time constant (τ, tau) was obtained. Multiple replicates were processed per condition, and average recovery curves (mean ± standard deviation) were computed. Recovery time constants from individual replicates were used for statistical comparisons across conditions using Welch's t-tests (scipy.stats). Plots of recovery curves and tau values were generated using Matplotlib.

Circularity determination

Coacervates were prepared using poly-(L-lysine)$_{100}$ and poly-(L-aspartic acid)$_{250}$, with the addition of 250nM, 500nM, 750nM, 1000nM, or 2500nM GFP-Tau protein stabilizer. Sulfo-Cyanine5 labeled poly-(L-lysine)$_{100}$ at a final dye concentration of 250nM was included to label the coacervate interiors. Labeling of the polypeptides with a sulfo-Cyanine5-NHS ester (Lumiprobe) was done following the manufacturer's protocols. The samples were imaged using confocal microscopy, and at least four locations within each sample were imaged to represent the sample population accurately.

The micrographs were duplicated using an ImageJ script, and the smooth function was applied to reduce graining. The images were then subjected to binary thresholding using the automatic built-in function of ImageJ, where the background was 0 and the coacervate interior 1. The resulting binary mask was subjected

to the watershed function to prevent nearby particles from overlapping. Next, the ImageJ plug-in "analyze particles" was used to detect particles with a minimum size of 100 pixels. This was done to exclude small droplets with insufficient pixels for accurate circularity determination and to avoid detecting out-of-focus droplets. Ellipsoids were fitted on the binary mask, and from the dimensions of the ellipsoids, the eccentricity (e) was determined as follows:

$$e = \sqrt{1 - \frac{b^2}{a^2}} \tag{1}$$

Where a equals half the length of the major axis, and b equals half the length of the minor axis. In the case of a perfect circle, $e = 0$. The eccentricity is here processed ($c = 1 - e$) such that a perfect circle has a circularity of 1.

The circularity threshold in Figure 1G was determined based on the average circularity observed at 2.5 μM GFP-Tau, adjusted by subtracting one standard deviation:

$$Stability\ threshold = \mu - \sigma \tag{2}$$

where μ is the mean circularity at 2.5 μM GFP-Tau, and σ is the corresponding standard deviation.

Cryo-TEM imaging

For cryo-TEM experiments, small coacervates were prepared in coacervate buffer (50mM HEPES pH 7.4, 150mM NaCl) by mixing 4uM of poly-(L-lysine)$_{100}$ (400uM positive charge) with 2.4uM of poly-(L-aspartic acid)$_{250}$ (600uM negative charge), resulting in a total monomeric charge concentration of 1mM. For the stabilized samples, 2.5uM of stabilizing protein was added before the induction of coacervation. The adjustments in the protocol resulted in smaller coacervates, which could be retained in the thin layer after the blotting process. Coacervate samples were immediately processed after preparation. All experiments were performed on the TU/e CryoTitan (Thermo Fisher Scientific) equipped with a field emission gun and autoloader and operated at 300 kV acceleration voltage in low-dose bright-field TEM mode. Samples for cryo-TEM were prepared by glow-discharging the grids (Quantifoil Cu grid with R 2/2 holey carbon films, Quantifoil Micro Tools GmbH, part of the SPT Life Sciences group) in a Cressington 208 carbon coater for 40 seconds. Then, 3 μL of the sample was pipetted on the grid and blotted in a Vitrobot MARK IV at room temperature and 100% humidity. The grid was blotted for 3 seconds (offset −3) and directly plunged and vitrified in liquid ethane. Cryo-TEM images were acquired with zero loss energy filtering mode (Gatan GIF 2002, 20 eV energy slit) on a CCD camera (Gatan model 794). Processing of all electron microscopy images was performed with ImageJ. Specifically, monolayer widths were quantified from the cryo-TEM micrographs shown in Figure 3 by drawing line profiles across the condensate interfaces using the line tool in ImageJ. For each droplet, multiple line profiles were taken at different points along the interface to account for potential variability and reduce user bias. The width of each interfacial feature was then measured using the "Measure" function in ImageJ, which reports the distance across the drawn line. While this approach is inherently manual, the relatively large number of measurements collected for each condition (as reflected in the scatter plot shown in Figure 3I) helped mitigate the effects of measurement uncertainty and variability in interface geometry. Plotting of the measured widths were performed using Origin.

Super-resolution microscopy

Single-molecule tracking photoactivated localization microscopy (sptPALM) images were acquired using a Nikon Eclipse Ti-E N-STORM system equipped with a Nikon 100x Apo total internal reflection fluorescence (TIRF) oil immersion objective (NA 1.49). Before acquisition, the TIRF angle was adjusted for HILO illumination, which allowed deeper imaging into the sample and visualization of diffusion of the proteins at the top interface with a low signal-to-noise ratio. Excitation and photoconversion of fluorescent proteins were performed by applying either the 488 nm excitation laser or the 405 nm and 561 nm excitation lasers within the MLC400B laser box (Agilent Technologies). For sptPALM acquisition, the power of the 405 nm laser was set up to 3% to stimulate sparse photoconversion of mEOS3.2-GST-Tau so that single proteins could be detected at the coacervate interface. The powers of the 488 and 561 nm laser were set to approximately 50-70%. The fluorescent signal was filtered by a quad-band polychroic mirror (97335 Nikon) and detected by an Ixon3 EMCCD (Andor) camera, resulting in a pixel size of 160 nm.

Coacervates were prepared as described for cryo-TEM using a total monomeric charge concentration of 1 mM and a total stabilizer concentration of 2.5 μM. By reducing the polymer concentration to 1 mM, smaller coacervates were prepared. This adjustment was required since the coacervates' refractive index caused interference during imaging.

For the experiments that validate protein docking at the coacervate interface, 25 nM of mEOS3.2-GST-Tau, 500 nM GFP-Tau, and 1975 nM of GST-Tau were used. During this experiment, the coacervates were first

imaged by exciting the GFP-Tau with the 488 nm laser. Then, sptPALM was performed by sparse photoconversion of mEOS3.2-GST-Tau with the 405 nm laser and excitation up to 70% with the 561 nm laser to allow single protein imaging. For the experiments involving the tracking of stabilizing proteins at the top of the coacervate interface, 50 nM of mEOS3.2-GST-Tau, 500 nM of GFP-Tau, and 1975 nM of GST-Tau were used. sptPALM imaging at the top of the coacervates allowed an area of approximately 1.5 μm^2 in which diffusion of the proteins could be tracked without going out of focus in the z-direction. For protein exchange experiments between the bulk and the interface, coacervates were prepared using 2000 nM of GST-Tau and 500 nM of GFP-Tau. Before adding mEOS3.2-GST-Tau, the same imaging procedure was used as with the docking experiment. After adding 100 nM of mEOS3.2-GST-Tau, first, the GFP-tagged proteins were imaged by using the 488 nm laser to confirm whether the coacervates were in focus. Second, the mEOS3.2-GST-Tau was photoconverted to allow single protein imaging. The acquisition time was set to 10 ms to determine the diffusion of fluorescent proteins on the coacervate interface and 20 ms when imaging the interface of coacervates to reconstruct the membrane.

For localization of fluorescent proteins, acquisitions were analyzed in ImageJ/Fiji (1.54f) software using the ThunderSTORM (1.3) plugin for detecting molecules and Detection of Molecules (v.1.2.5) to link particles to tracks. The maximum distance for a detected molecule to move was set to two pixels, and a maximum linking gap of one frame. Tracks were visualized in Origin 2020.

2. SUPPLEMENTARY FIGURES

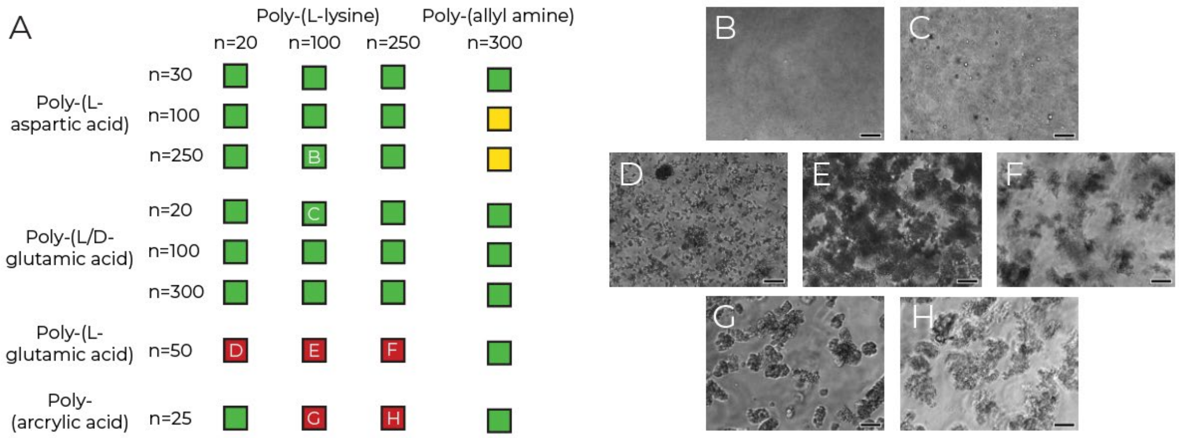

Supplementary Figure S1: Phase separating behavior of various polyelectrolytes, as determined by brightfield microscopy. (**A**) For the screening process, polymer mixtures were prepared at a total monomeric charge concentration of 8mM and five different positive-to-negative charge ratios (3:7, 4:7, 5:5, 6:4, 7:3). Green color indicates phase-separated combinations under at least one experimental condition. Yellow indicates combinations that did not phase separate under any of the conditions. Red indicates incompatible polyelectrolyte combinations that were found to form solid aggregate structures. Illustrative brightfield images of the polyelectrolyte combinations marked in (**A**) are shown in (**B-I**). Images (**B-C**) are representative images that were classified as phase separation. Images (**D-H**) represent different types of aggregation behaviors. Scalebars: 50µm.

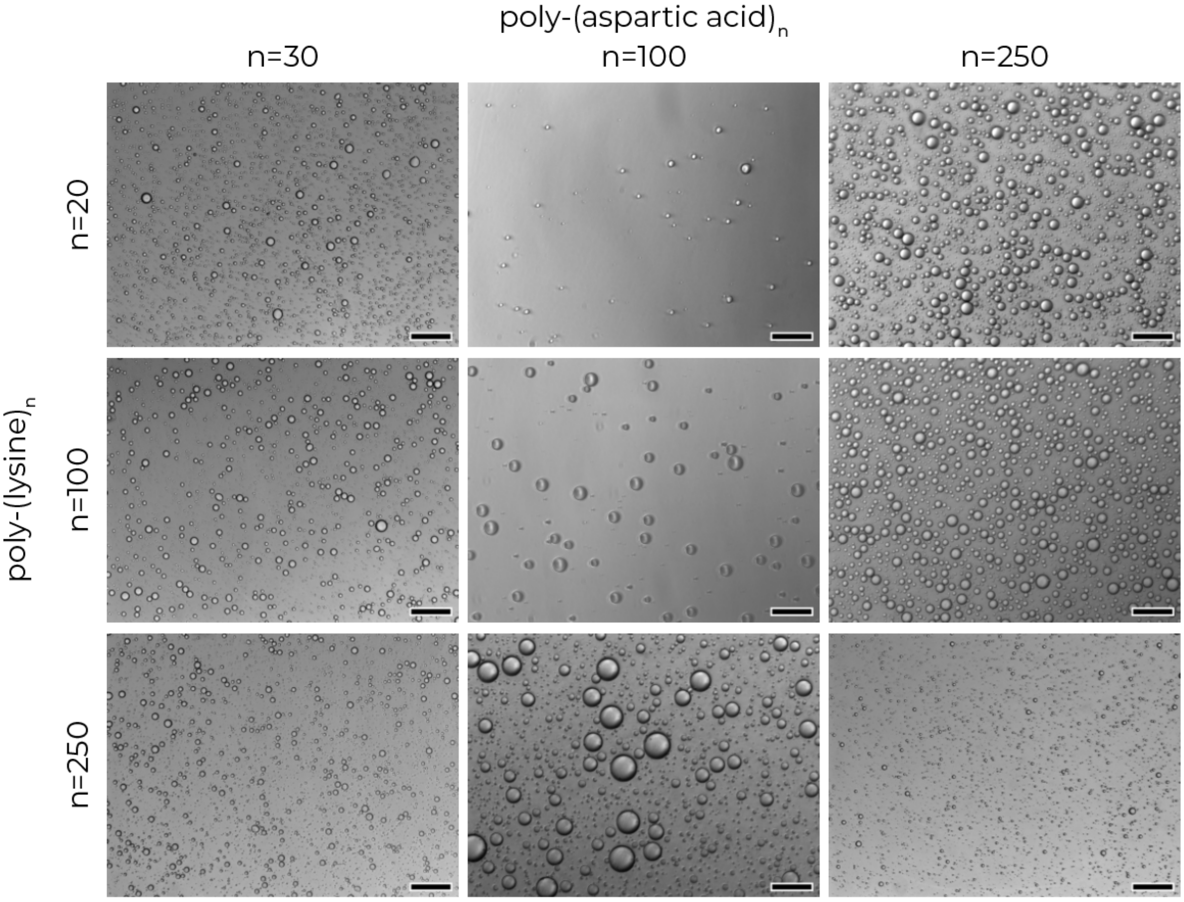

Supplementary Figure S2: Representative brightfield images of poly-(L-lysine)/poly-(L-aspartic acid) coacervates imaged on BSA-coated glass slides to minimize substrate-induced wetting and preserve droplet morphology. All samples were formulated using a 40:60 molar ratio of lysine to aspartic acid monomer units, combining poly-(L-lysine) chains of 20, 100, and 250 residues with poly-(L-aspartic acid) chains of 30, 100, and 250 residues. These experiments were performed under passivated conditions to confirm that coacervates form across this matrix of polypeptide combinations, independent of stabilization. Under these passivated conditions, coacervates remained intact overnight, enabling reliable visualization of droplet formation across a wide range of conditions. The resulting droplet sizes and distributions are consistent with those observed in stabilized systems (see Supplementary Figures S7–S8), highlighting the impact of surface interactions on condensate stability and appearance. Scale bars: 50 µm.

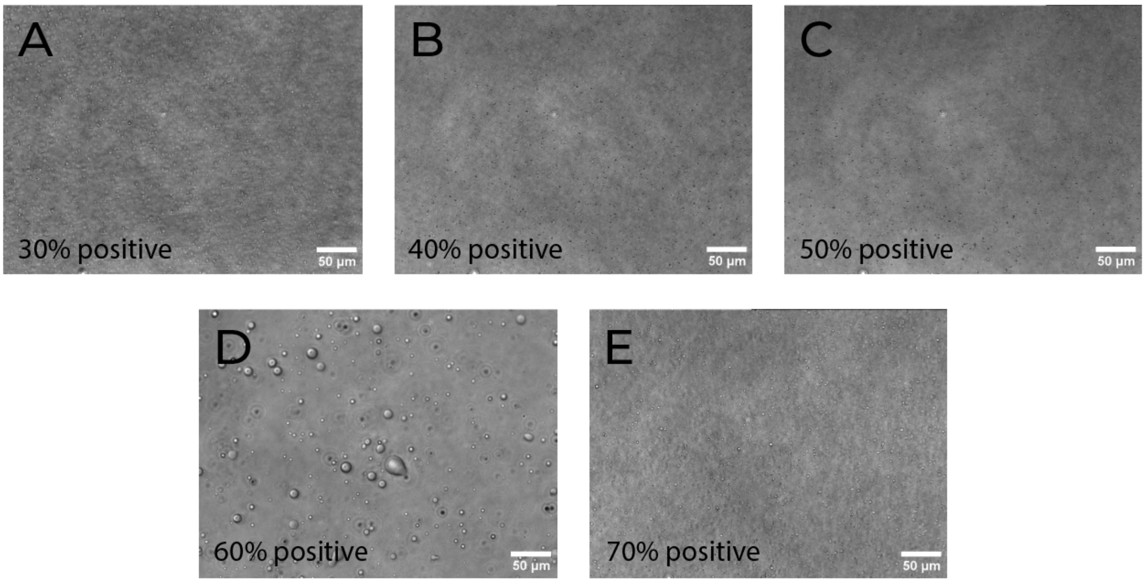

Supplementary Figure S3: Brightfield micrographs of coacervates formed from poly-(L-lysine)$_{100}$ and poly-(L-aspartic acid)$_{250}$ in the absence of protein stabilizer. Samples were prepared at a total monomeric charge concentration of 8mM with varying positive-to-negative charge ratios. (**A**) 3:7 (positive:negative), (**B**) 2:3, (**C**) 1:1, (**D**) 3:2, (**E**) 7:3. Because no stabilizing protein was used, these coacervates are unstable and readily dissolve, wet, or fuse upon contact with the well plate. The small droplet size observed is largely due to rapid disintegration of larger droplets upon substrate contact, resulting in a bias toward smaller or fewer visible droplets by the time of imaging. Micrographs were taken 5 min after coacervate production. Scalebars: 50µm.

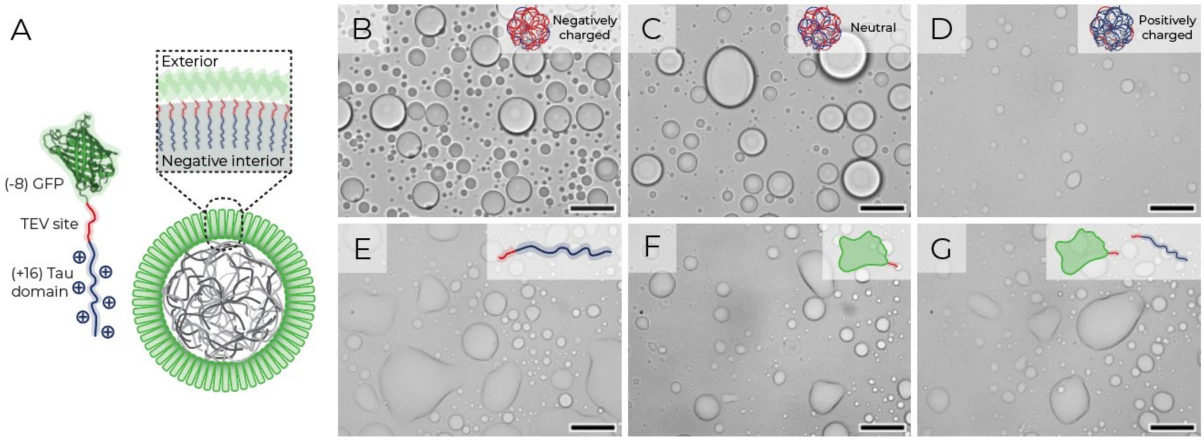

Supplementary Figure S4: Electrostatic interactions and buoyancy drive coacervate stabilization. (**A**) Schematic of Tau–GFP, with Tau aligning toward the coacervate interior and GFP toward the dilute phase. (**B–D**) Brightfield images of negatively charged (**B**), neutral (**C**), and positively charged (**D**) coacervates with Tau–GFP. (**E–G**) Coacervates in presence of Tau (**E**), GFP (**F**), or both (**G**). Scalebars: 30 µm.

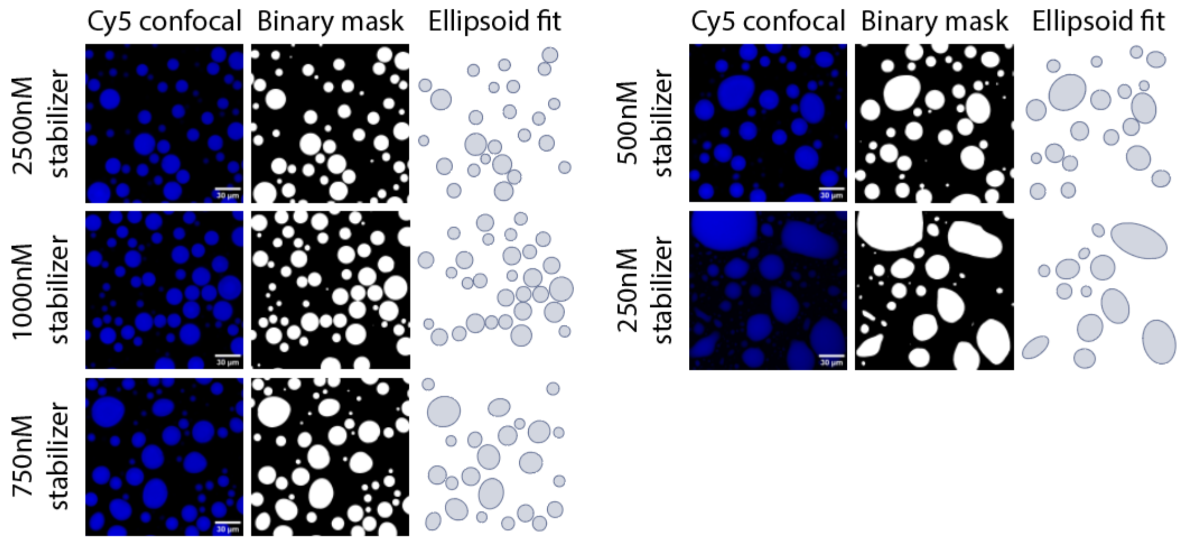

Supplementary Figure S5: Circularity determination from confocal micrographs. This figure shows the process used to determine the circularity of coacervates at different stabilizer concentrations, contributing to the circularity plot in Figure 1G. To prepare coacervates, poly-(L-lysine)$_{100}$ and poly-(L-aspartic acid)$_{250}$ were used. A portion of poly-(L-lysine)$_{100}$ was labeled with NHS-SulfoCy5 to enable confocal imaging. The (bulk) concentration of Cy5 (blue) used was 250nM, and representative confocal micrographs for each stabilizer concentration are shown in the first columns. Confocal images were processed in ImageJ to a binary image, as shown in the second column. The third column shows ellipsoids fitted to the binary images. From the ellipsoid dimensions, the circularity (c) is calculated as described in the Methods, Eq. 1. Scalebars: 30μm.

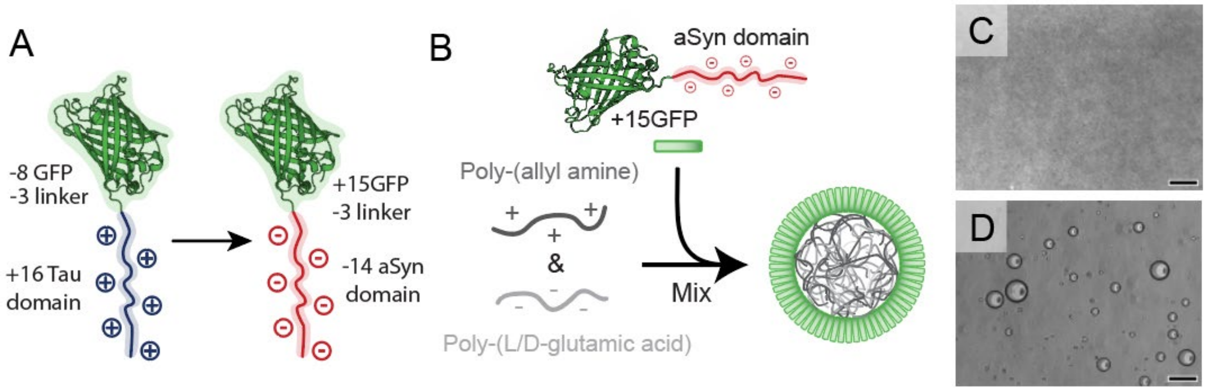

Supplementary Figure S6: Protein engineering of a coacervate stabilizer for positively charged coacervates. (**A**) The positively charged tau domain (+16) was substituted with a negatively charged alpha-synuclein protein domain (-14), which is attracted to positively charged coacervates through electrostatic forces. In the GFP-Tau fusion protein, the GFP protein (-8) plays a crucial role in preventing the internalization of the protein into negative coacervates by providing electrostatic and steric buoyancy. To achieve a similar effect with the alpha-synuclein fusion protein for positive coacervates, GFP was substituted with +15GFP, resulting in the final +15GFP-aSyn protein. (**B**) Stabilized positively charged coacervates were prepared by mixing poly-(allyl amine)$_{300}$ with poly-(L/D-glutamic acid)$_{100}$ in the presence of +15GFP-aSyn at a positive-to-negative charge ratio of 3:2 and a total monomeric charge ratio of 8mM. The coacervates formed in the absence of stabilizing protein are shown in the brightfield image in panel (**C**), while the coacervates formed in the presence of 5uM of stabilizing protein are shown in the brightfield image in panel (**D**). Scalebars: 50µm.

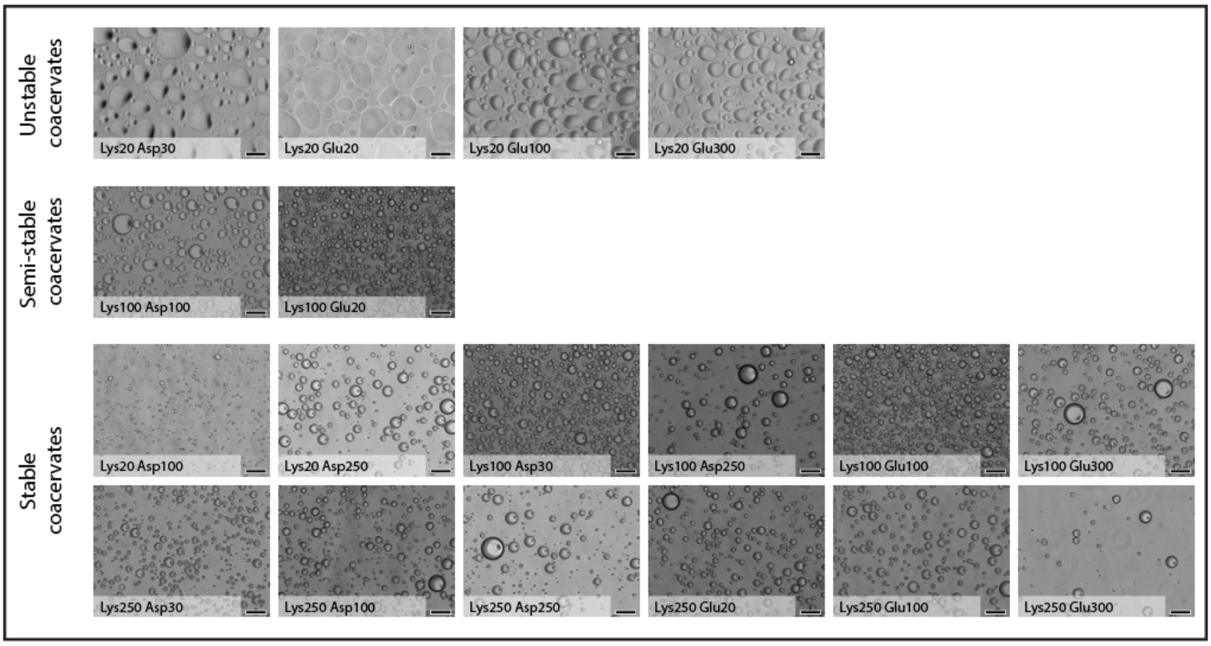

Supplementary Figure S7: The stabilizing effect of 2.5µM GST-Tau against a library of phase-separating polypeptides. The presented brightfield images were used to determine the stability classification shown in Figure 3E. The images show specific polypeptide compositions and are grouped based on stability, determined by the metrics described in the main text. Negative coacervate samples were created by mixing a 2:3 monomer ratio of the positive-to-negative polypeptide with a total monomeric charge concentration of 8 mM. Scalebars: 50µm.

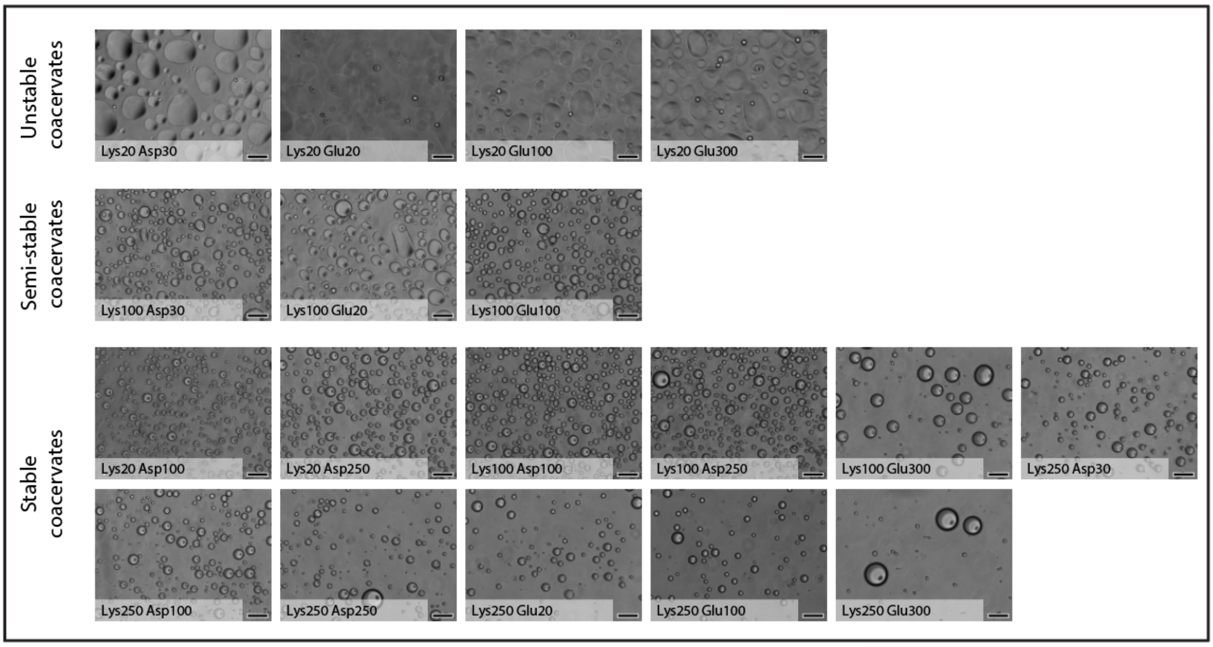

Supplementary Figure S8: The stabilizing effect of 2.5μM GFP-Tau against a library of phase-separating polypeptides. The presented brightfield images were used to determine the stability classification shown in Figure 3F. The images show specific polypeptide compositions and are grouped based on stability, which is determined by the metrics described in the main text. Negative coacervate samples were created by mixing a 2:3 monomer ratio of the positive-to-negative polypeptide with a total monomeric charge concentration of 8mM. Scalebars: 50μm.

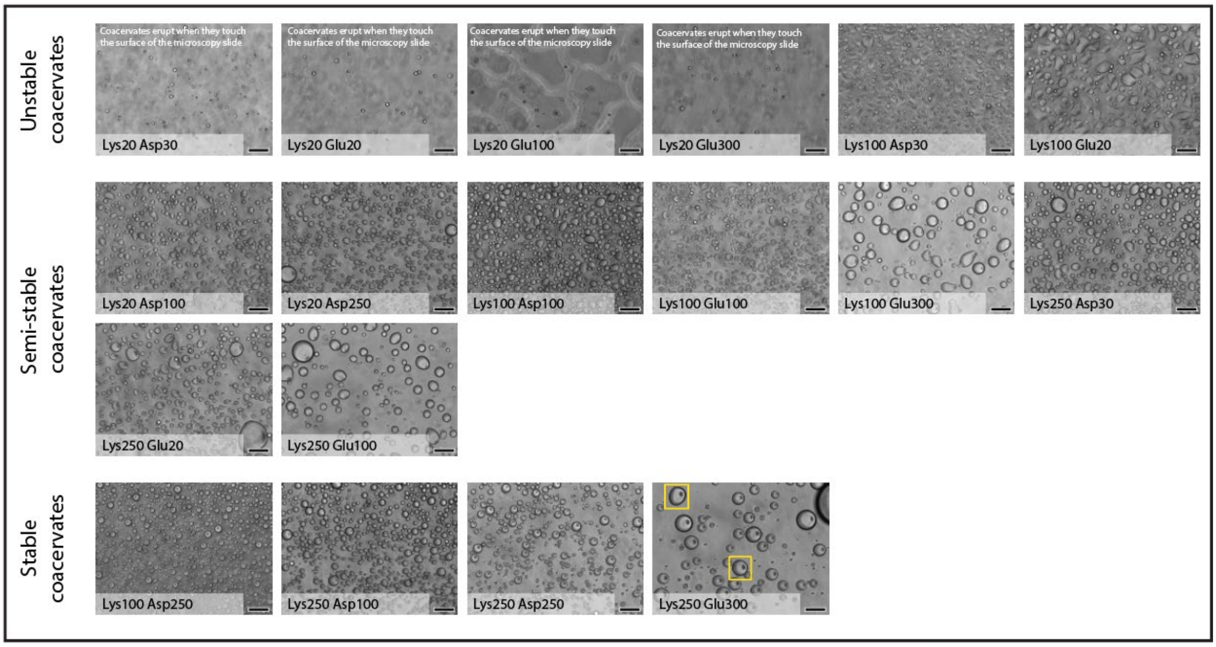

Supplementary Figure S9: The stabilizing effect of 2.5μM muGFP-Tau against a library of phase-separating polypeptides. The presented brightfield images were used to determine the stability classification shown in Figure 3G. The images show specific polypeptide compositions and are grouped based on stability, which is determined by the metrics described in the main text. Negative coacervate samples were created by mixing a 2:3 monomer ratio of the positive-to-negative polypeptide with a total monomeric charge concentration of 8mM. Yellow squares represent ambiguous coacervates, for which a clear classification could not be determined. Due to the convincing stability of the remaining bulk coacervates, the entirety of the sample was classified as stable. Scalebars are 50μm.

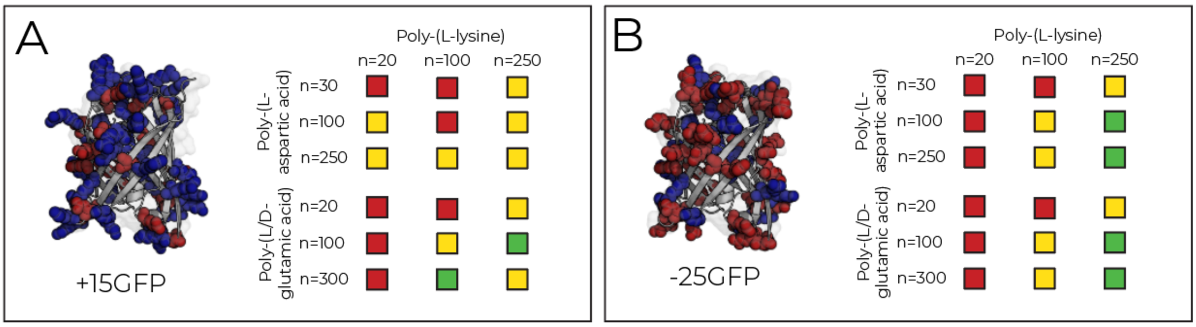

Supplementary Figure S10: Suppressing dimerization through supercharging the buoyancy group reduces coacervate stability. Dimerization was supressed by replacing GFP in GFP-Tau with (**A**) +15GFP to produce +15GFP-Tau or (**B**) -25GFP to produce -25GFP-Tau. Both fusion proteins were screened for their stabilizing effect on a library of 18 phase-separating polypeptide combinations. Using the same metrics as in Figure 3, green, yellow, and red indicate stable, semi-stable, and unstable formulations, respectively. The presented structures for +15GFP (**A**) and -25GFP (**B**) are AlphaFold predictions, where positive amino acids are shown as blue spheres, negative amino acids as red spheres, and the protein structure as a grey cartoon.

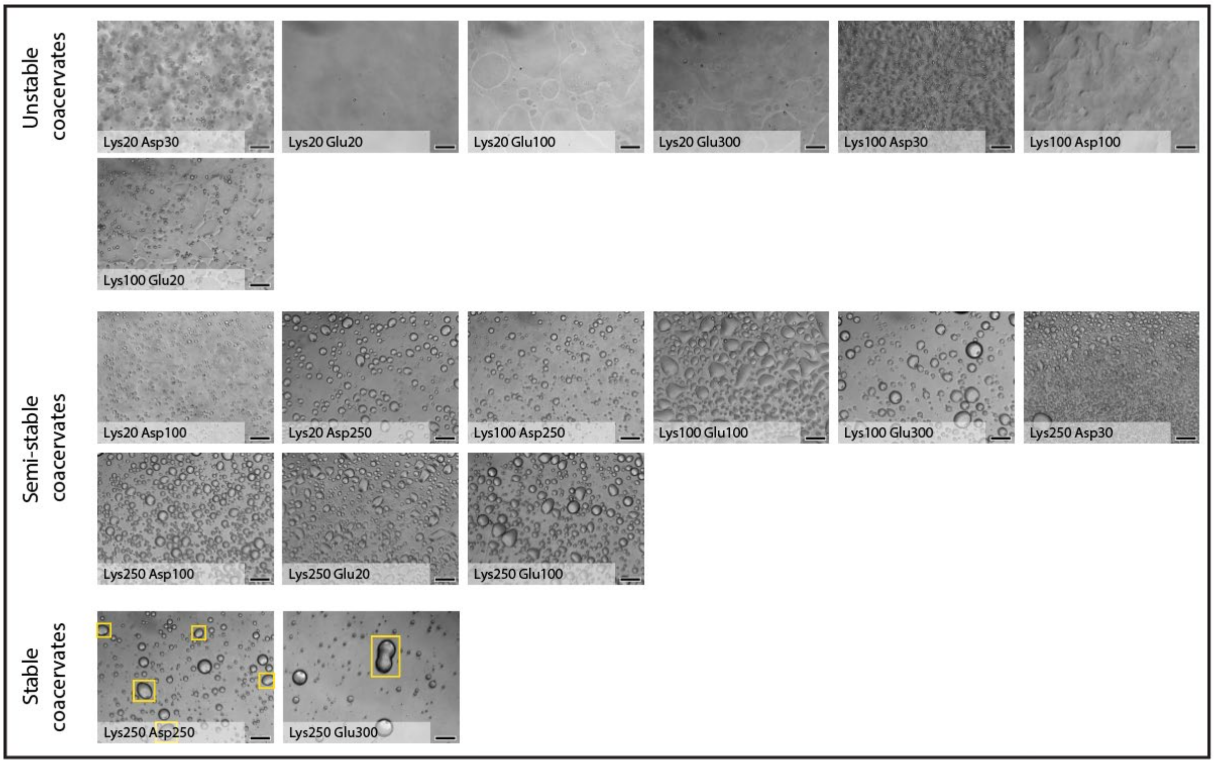

Supplementary Figure S11: The stabilizing effect of 2.5µM +15GFP-Tau against a library of phase-separating polypeptides. The presented brightfield images were used to determine the stability classification shown in Supplementary Figure S10. The images show specific polypeptide compositions and are grouped based on stability, which is determined by the metrics described in the main text. Negative coacervate samples were created by mixing a 2:3 monomer ratio of the positive-to-negative polypeptide with a total monomeric charge concentration of 8mM. The yellow squares represent ambiguous coacervates, for which a clear classification could not be determined. Due to the convincing stability of the remaining bulk coacervates, the entirety of the sample was classified as stable. Scalebars: 50µm.

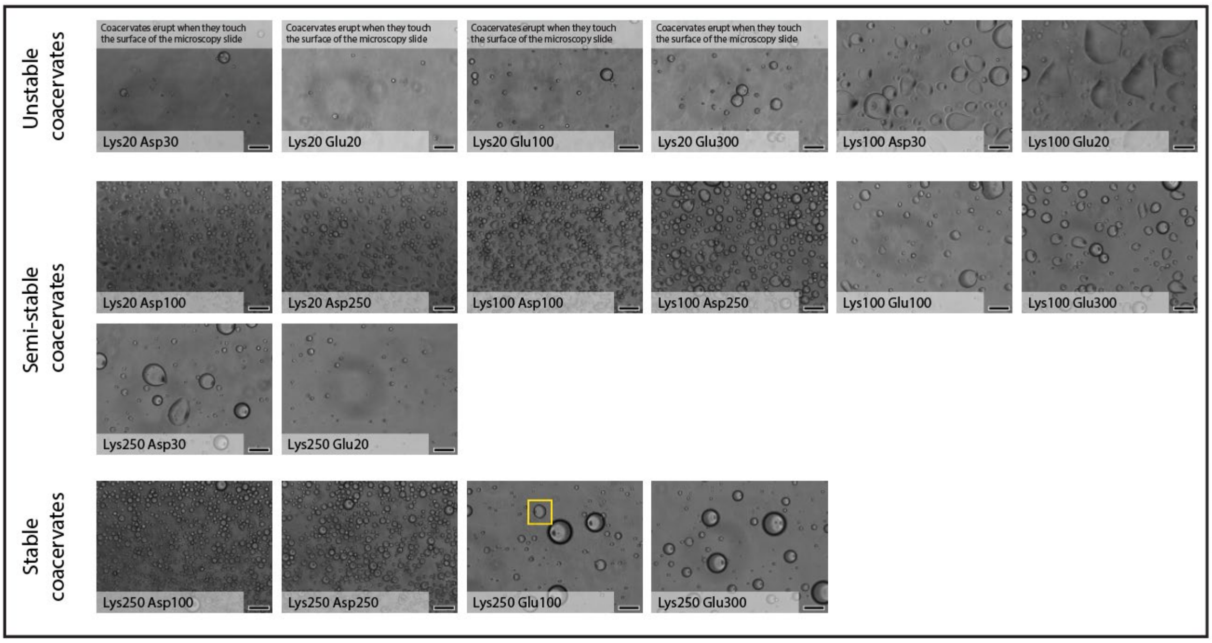

Supplementary Figure S12: The stabilizing effect of 2.5µM -25GFP-Tau against a library of phase-separating polypeptides. The presented brightfield images were used to determine the stability classification shown in Supplementary Figure S10. The images show specific polypeptide compositions and are grouped based on stability, which is determined by the metrics described in the main text. Negative coacervate samples were created by mixing a 2:3 monomer ratio of the positive-to-negative polypeptide with a total monomeric charge concentration of 8mM. The yellow square represents ambiguous coacervates, for which a clear classification could not be determined. Due to the convincing stability of the remaining bulk coacervates, the entirety of the sample was classified as stable. Scalebars: 50µm.

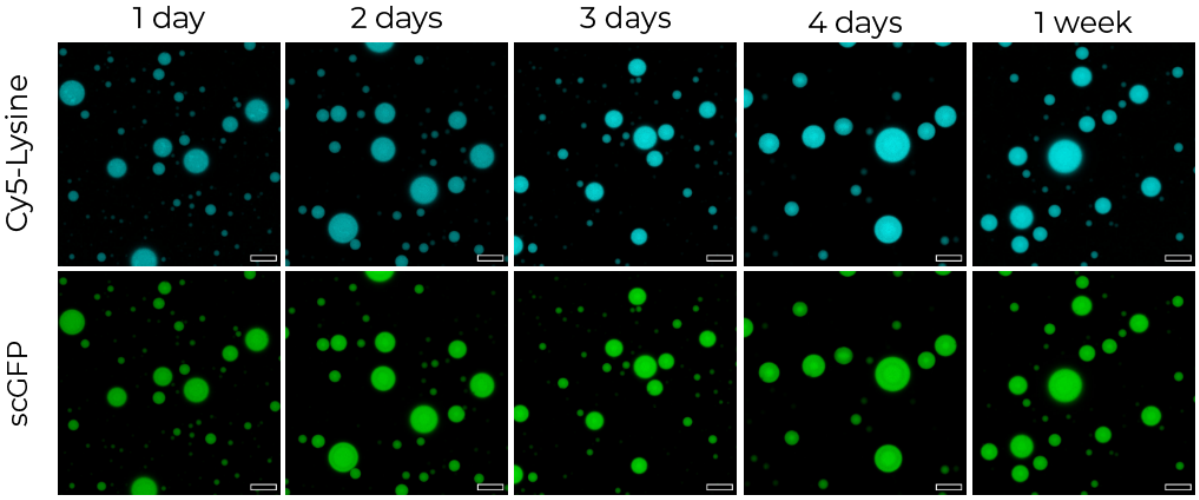

Supplementary Figure S13: Protein-stabilized coacervates remain morphologically stable over extended time periods. Coacervates were formed from poly-(L-lysine)$_{100}$ and poly-(L-aspartic acid)$_{250}$, stabilized with 2.5 µM GST-Tau. Cy5-labeled poly-(L-lysine)$_{100}$ (100 nM dye) and supercharged GFP (+36GFP, 250 nM) were included to visualize coacervate structure and cargo retention. Fluorescence images of the same sample acquired over the course of one week (1, 2, 3, 4, and 7 days) show that droplets remain intact and retain both components without visible spreading, wetting, or fusion. The top row shows Cy5-lysine signal; the bottom row shows GFP signal. Coacervate samples were prepared by mixing a 2:3 monomer ratio of the positive-to-negative polypeptides, at a total monomeric charge concentration of 8 mM. Scale bars: 30 µm.

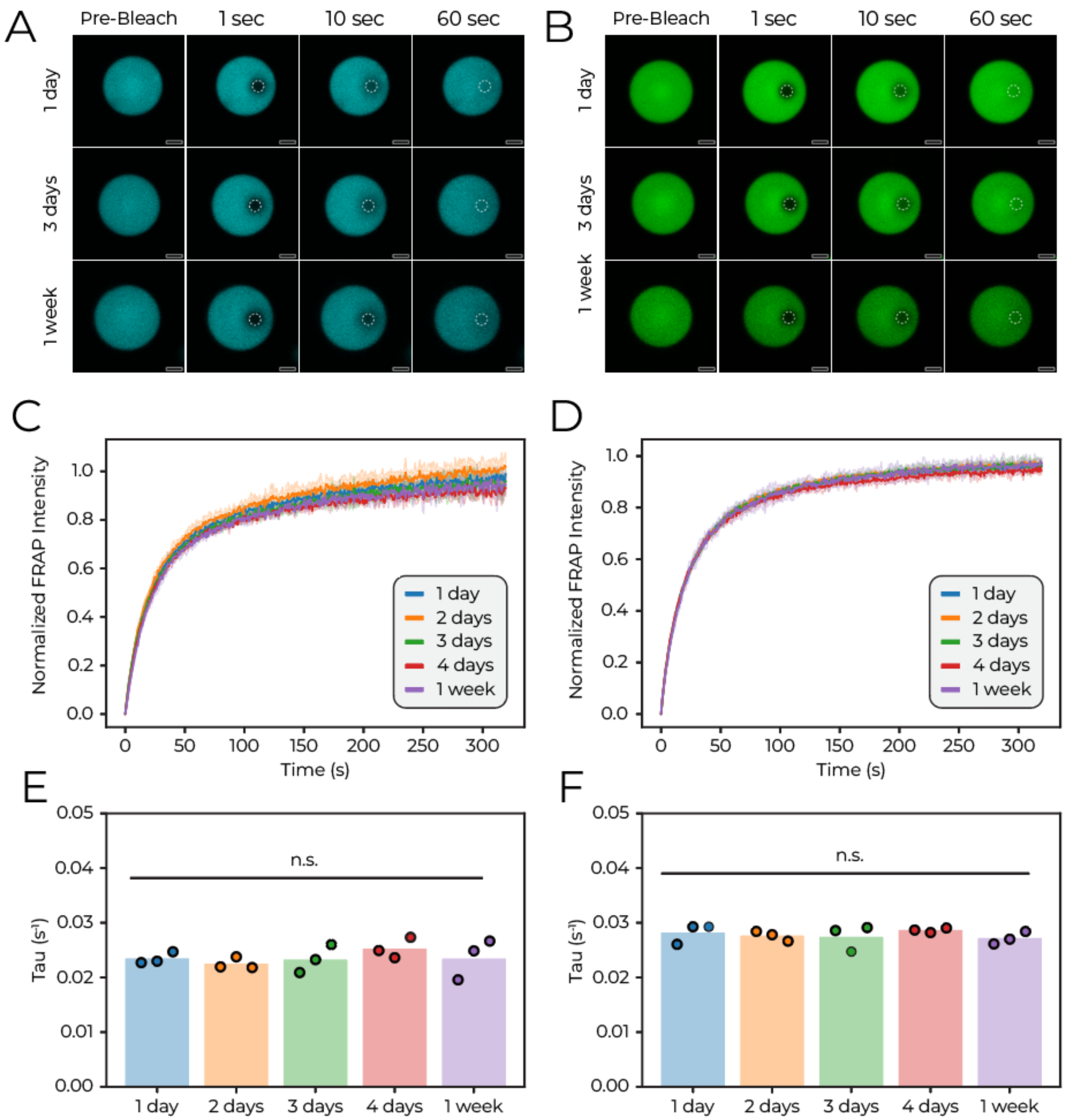

Supplementary Figure S14: The internal molecular dynamics of GST-Tau-stabilized coacervates remains unchanged over time. (**A–B**) Representative confocal FRAP micrographs at 1 day, 3 days, and 1 week for (**A**) Cy5-labeled poly-(L-lysine)$_{100}$ (100 nM dye) and (**B**) supercharged GFP (+36GFP, 250 nM), used as representative internal components. The white dotted circle marks the bleach region of 4µm in diameter. Coacervates were formed from poly-(L-lysine)$_{100}$ and poly-(L-aspartic acid)$_{250}$ at a monomer ratio of 40% lysine to 60% aspartic acid, stabilized with 2.5 µM GST-Tau. (**C–D**) Normalized FRAP recovery curves at five time points (1 to 7 days) for (**C**) Cy5-labeled poly-(L-lysine)$_{100}$ and (**D**) +36GFP demonstrate consistent recovery kinetics over time. (**E–F**) Extracted τ (tau) values from exponential fits for (**E**) Cy5-labeled poly-(L-lysine) and (**F**) +36GFP show no statistically significant differences across time points ($p > 0.05$ for all comparisons, pairwise Welch's t-tests). Each bar represents biological triplicates from independently prepared samples, with individual measurements shown as dots.

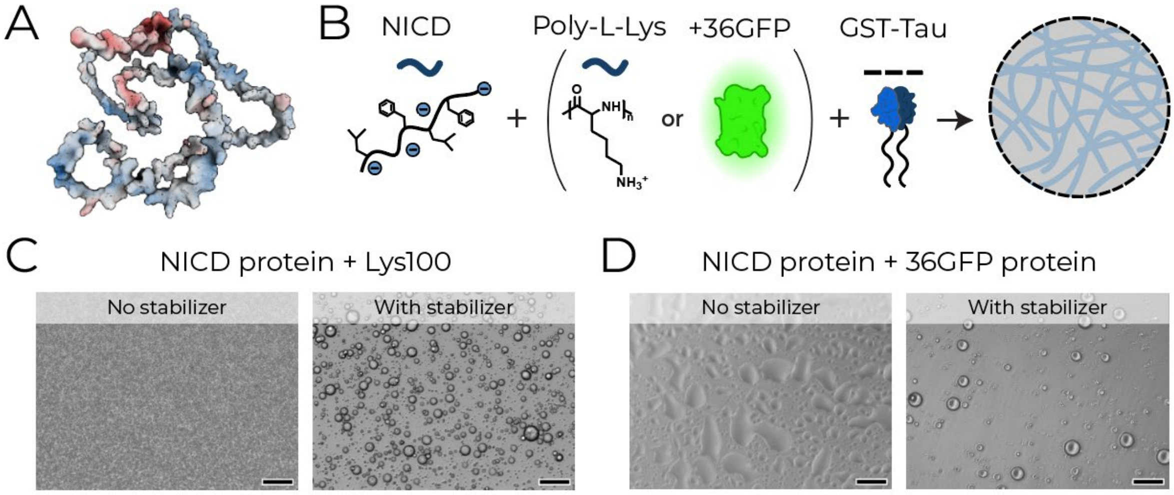

Supplementary Figure S15: Stabilization of NICD-based coacervates using the GST-Tau fusion protein. (**A**) AlphaFold-prediction structure of the intrinsically disordered Nephrin Intracellular Domain (NICD), colored by electrostatic surface potential (red: positive residues, blue: negative residues). (**B**) Schematic representation of a coacervate system in which the negatively charged NICD protein undergoes phase separation with cationic components (poly-(L-lysine) or +36GFP), forming coacervates that can be stabilized by the surface-active GST-Tau protein. (**C–D**) Brightfield images of coacervates formed by NICD with either poly-(L-lysine)$_{100}$ (**C**) or the positively supercharged globular protein +36GFP (**D**), in the absence or presence of 2.5 µM GST-Tau stabilizer. Without stabilizing protein, condensates display limited morphological stability, while the addition of GST-Tau preserves droplet integrity overnight. These examples illustrate the applicability of GST-Tau to coacervates formed from components with distinct structural and charge properties. Scale bars: 50 µm.

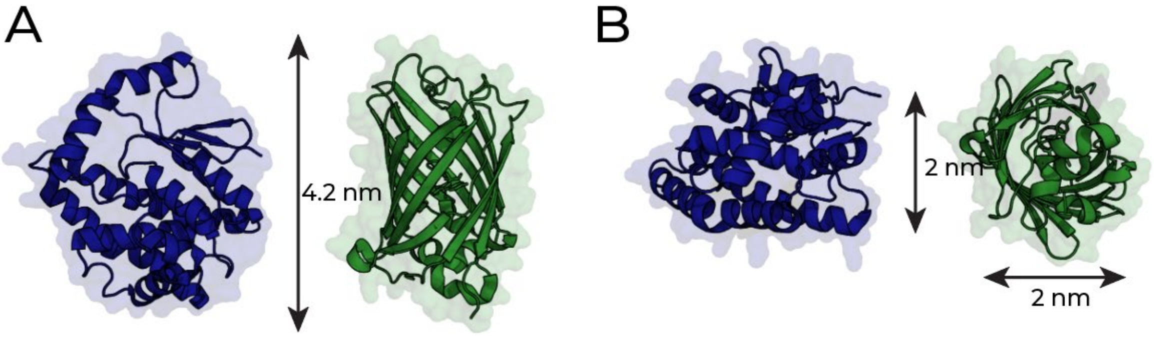

Supplementary Figure S16: Size comparison of Green Fluorescent Protein (GFP) and Glutathione S-transferase (GST) based on their crystal structures. (**A**) Front- or (**B**) top-view of the GST protein (PDB: 1PKW; blue) and GFP (PDB: 5NHN; green). Both proteins are shown at the same scale, allowing their dimensions to be directly compared. The arrows indicate the dimensions of GFP, as reported by Hink *et al.* (2000). According to their crystal structures, the sizes of GFP and GST are comparable.

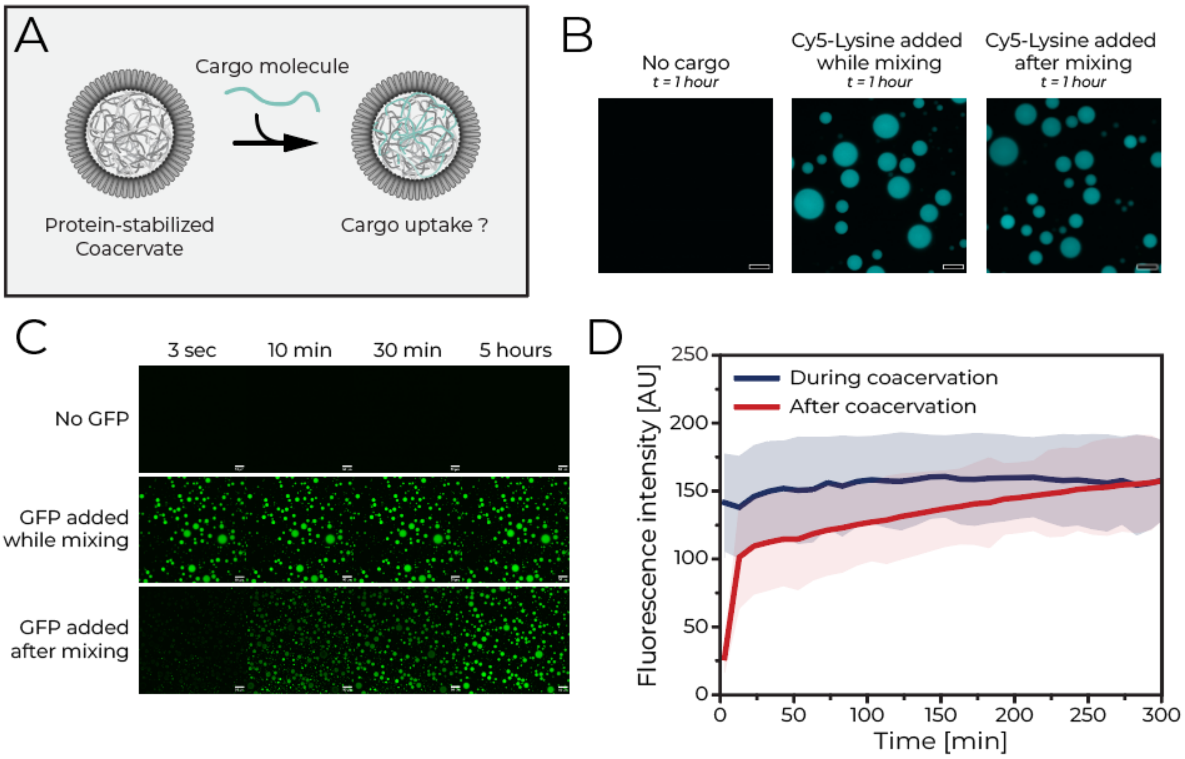

Supplementary Figure S17: Surface-stabilized coacervates remain permeable and support post-formation uptake of cargo molecules. (**A**) Schematic illustration of a cargo uptake assay to test whether externally added molecules can enter pre-formed, protein-stabilized coacervates. (**B**) Confocal micrographs of coacervates formed from poly-(L-lysine)$_{20}$ and poly-(L-aspartic acid)$_{250}$, stabilized with 2.5 µM GST-Tau. Cy5-labeled poly-(L-lysine)$_{20}$ (100 nM dye) was added either during or after coacervate formation. In both cases, strong accumulation of the cargo is observed within the coacervate droplets one hour after addition. (**C**) Confocal time-lapse imaging of supercharged GFP (+36GFP, 250 nM) uptake into the same coacervate formulation, added either during or after droplet formation. Fluorescence intensity within the coacervates increases rapidly, particularly within the first 10 minutes. (**D**) Quantification of mean fluorescence intensity over time confirms the rapid partitioning of GFP into the coacervate phase. Shaded regions represent the standard deviation of particle intensities within a single imaging position. All coacervates were prepared using a 2:3 monomer ratio of lysine to aspartic acid. Scale bars: 30 µm.

3. PROTEIN DESIGN AND PROPERTIES

Supplementary Table 1: Sequences and parameters of the proteins used in this study. Parameters were calculated using the online ProtParam tool (ExPASy)

Construct name	Sequence	Molecular weight (Da)	Isoelectric point	Charge at pH 7.4
**GFP-Tau**				
GFP-TEV-Tau-Strep	MVKMGASKGEELFTGVVPILVELDGDVNGHKF<br>SVSGEGEGDATYGKLTLKFICTTGKLPVPWPTLV<br>TTFSYGVQCFSRYPDHMKQHDFFKSAMPEGY<br>VQERTIFFKDDGNYKTRAEVKFEGDTLVNRIELK<br>GIDFKEDGNILGHKLEYNYNSHNVYIMADKQK<br>NGIKVNFKIRHNIEDGSVQLADHYQQNTPIGD<br>GPVLLPDNHYLSTQSALSKDPNEKRDHMVLLE<br>FVTAAGITHGMDELYKTLPETGDYDIPTTENLYF<br>QGGSPGTPGSRSRTPSLPTPPTREPKKVAVVRT<br>PPKSPSSAKSRLQTAPVPMPDLKNVKSKIGSTE<br>NLKHQPGGGKVQIINKKLDLSNVQSKCGSKD<br>NIKHVPGGGSVQIVYKPVDLSKVTSKCGSLGNI<br>HHKPGGGQVEVKSEGGSWSHPQFEK	45692	8.71	+5
**GFP-Tau after TEV cleavage**				
GFP-TEV	MVKMGASKGEELFTGVVPILVELDGDVNGHKF<br>SVSGEGEGDATYGKLTLKFICTTGKLPVPWPTLV<br>TTFSYGVQCFSRYPDHMKQHDFFKSAMPEGY<br>VQERTIFFKDDGNYKTRAEVKFEGDTLVNRIELK<br>GIDFKEDGNILGHKLEYNYNSHNVYIMADKQK<br>NGIKVNFKIRHNIEDGSVQLADHYQQNTPIGD<br>GPVLLPDNHYLSTQSALSKDPNEKRDHMVLLE<br>FVTAAGITHGMDELYKTLPETGDYDIPTTENLYF<br>Q	29572	5.36	-11
TEV-Tau-Strep	GGSPGTPGSRSRTPSLPTPPTREPKKVAVVRTP<br>PKSPSSAKSRLQTAPVPMPDLKNVKSKIGSTEN<br>LKHQPGGGKVQIINKKLDLSNVQSKCGSKDNI<br>KHVPGGGSVQIVYKPVDLSKVTSKCGSLGNIH<br>HKPGGGQVEVKSEGGSWSHPQFEK	16137	10.21	+16
**GST-Tau:**				
GST-TEV-Tau-Strep	MGSPILGYWKIKGLVQPTRLLLEYLEEKYEEHLY<br>ERDEGDKWRNKKFELGLEFPNLPYYIDGDVKL<br>TQSMAIIRYIADKHNMLGGCPKERAEISMLEGA<br>VLDIRYGVSRIAYSKDFETLKVDFLSKLPEMLKM<br>FEDRLCHKTYLNGDHVTHPDFMLYDALDVVLY<br>MDPMCLDAFPKLVCFKKRIEAIPQIDKYLKSSKY<br>IAWPLQGWQATFGGGDHPPKDYDIPTTENLYF<br>QGGSPGTPGSRSRTPSLPTPPTREPKKVAVVRT<br>PPKSPSSAKSRLQTAPVPMPDLKNVKSKIGSTE<br>NLKHQPGGGKVQIINKKLDLSNVQSKCGSKD<br>NIKHVPGGGSVQIVYKPVDLSKVTSKCGSLGNI<br>HHKPGGGQVEVKSEGGSWSHPQFEK	43276	9.05	+10
**muGFP-Tau:**				

muGFP-TEV-Tau-Strep	MGSSKGEELFTGVVPILVELDGDVNGHKFSVR<br>GEGEGDATNGKLTLKFICTTGKLPVPWPTLVTTL<br>TYGVLCFSRYPDHMKRHDFFKSAMPEGYVQE<br>RTISFKDDGTYKTRAEVKFEGDTLVNRIELKGIDF<br>KEDGNILGHKLEYNFNSHNVYITADKQKNGIKA<br>YFKIRHNVEDGSVQLADHYQQNTPIGDGPVLL<br>PDNHYLSTQSVLSKDPNEKRDHMVLLEDVTAA<br>GITHGMDELYKDYDIPTTENLYFQGGSPGTPGS<br>RSRTPSLPTPPTREPKKVAVVRTPPKSPSSAKSR<br>LQTAPVPMPDLKNVKSKIGSTENLKHQPGGGK<br>VQIINKKLDLSNVQSKCGSKDNIKHVPGGGSV<br>QIVYKPVDLSKVTSKCGSLGNIHHKPGGGQVE<br>VKSEGGSWSHPQFEK	44648	8.84	+6
**+15GFP-Tau:**				
+15GFP-TEV-Tau-Strep	MGASKGERLFTGVVPILVELDGDVNGHKFSVR<br>GEGEGDATRGKLTLKFICTTGKLPVPWPTLVTTL<br>TYGVQCFSRYPKHMKRHDFFKSAMPEGYVQE<br>RTISFKKDGTYKTRAEVKFEGRTLVNRIELKGRD<br>FKEKGNILGHKLEYNFNSHNVYITADKRKNGIK<br>ANFKIRHNVKDGSVQLADHYQQNTPIGRGPVL<br>LPRNHYLSTRSALSKDPKEKRDHMVLLEFVTAA<br>GITHGMDELYKDYDIPTTENLYFQGGSPGTPGS<br>RSRTPSLPTPPTREPKKVAVVRTPPKSPSSAKSR<br>LQTAPVPMPDLKNVKSKIGSTENLKHQPGGGK<br>VQIINKKLDLSNVQSKCGSKDNIKHVPGGGSV<br>QIVYKPVDLSKVTSKCGSLGNIHHKPGGGQVE<br>VKSEGGSWSHPQFEK	44945	9.84	+28
**-25GFP-Tau:**				
-25GFP-TEV-Tau-Strep	MGASKGEELFTGVVPILVELDGDVNGHEFSVR<br>GEGEGDATEGELTLKFICTTGELPVPWPTLVTTL<br>TYGVQCFSRYPDHMKQHDFFKSAMPEGYVQE<br>RTISFKDDGTYKTRAEVKFEGDTLVNRIELKGIDF<br>KEDGNILGHKLEYNFNSHDVYITADKQENGIKA<br>EFEIRHNVEDGSVQLADHYQQNTPIGDGPVLL<br>PDDHYLSTESALSKDPNEDRDHMVLLEFVTAA<br>GIDHGMDELYKDYDIPTTENLYFQGGSPGTPG<br>SRSRTPSLPTPPTREPKKVAVVRTPPKSPSSAKS<br>RLQTAPVPMPDLKNVKSKIGSTENLKHQPGGG<br>KVQIINKKLDLSNVQSKCGSKDNIKHVPGGGS<br>VQIVYKPVDLSKVTSKCGSLGNIHHKPGGGQV<br>EVKSEGGSWSHPQFEK	44612	5.70	-12
**mEOS3.2-GST-Tau:**				
mEOS3.2-GST-TEV-Tau-Strep	MGSAIKPDMKIKLRMEGNVNGHHFVIDGDGT<br>GKPFEGKQSMDLEVKEGGPLPFAFDILTTAFHY<br>GNRVFAKYPDNIQDYFKQSFPKGYSWERSLTF<br>EDGGICNARNDITMEGDTFYNKVRFYGTNFPA<br>NGPVMQKKTLKWEPSTEKMYVRDGVLTGDIE<br>MALLLEGNAHYRCDFRTTYKAKEKGVKLPGAH<br>FVDHCIEILSHDKDYNKVKLYEHAVAHSGLPDN<br>ARRGGSGGSSPILGYWKIKGLVQPTRLLLEYLE<br>EKYEEHLYERDEGDKWRNKKFELGLEFPNLPY<br>YIDGDVKLTQSMAIIRYIADKHNMLGGCPKERA<br>EISMLEGAVLDIRYGVSRIAYSKDFETLKVDFLSK<br>LPEMLKMFEDRLCHKTYLNGDHVTHPDFMLY<br>DALDVVLYMDPMCLDAFPKLVCFKKRIEAIPQI	69273	8.81	+10

	DKYLKSSKYIAWPLQGWQATFGGGDHPPKDYDIPTTENLYFQGGSPGTPGSRSRTPSLPTPPTREPKKVAVVRTPPKSPSSAKSRLQTAPVPMPDLKNVKSKIGSTENLKHQPGGGKVQIINKKLDLSNVQSKCGSKDNIKHVPGGGSVQIVYKPVDLSKVTSKCGSLGNIHHKPGGGQVEVKSEGGSWSHPQFEK			
**+15GFP-aSyn**				
+15GFP-TEV-aSyn-Strep	MGASKGERLFTGVVPILVELDGDVNGHKFSVRGEGEGDATRGKLTLKFICTTGKLPVPWPTLVTTLTYGVQCFSRYPKHMKRHDFFKSAMPEGYVQERTISFKKDGTYKTRAEVKFEGRTLVNRIELKGRDFKEKGNILGHKLEYNFNSHNVYITADKRKNGIKANFKIRHNVKDGSVQLADHYQQNTPIGRGPVLLPRNHYLSTRSALSKDPKEKRDHMVLLEFVTAAGITHGMDELYKDYDIPTTENLYFQGDQLGKNEEGAPQEGILEDMPVDPDNEAYEMPSEEGYQDYEPEAGGSWSHPQFEK	34936	6.61	-2
**+36GFP**				
+36GFP-TEV-His	MVKMGASKGERLFRGKVPILVELKGDVNGHKFSVRGKGKGDATRGKLTLKFICTTGKLPVPWPTLVTTLTYGVQCFSRYPKHMKRHDFFKSAMPKGYVQERTISFKKDGKYKTRAEVKFEGRTLVNRIKLKGRDFKEKGNILGHKLRYNFNSHKVYITADKRKNGIKAKFKIRHNVKDGSVQLADHYQQNTPIGRGPVLLPRNHYLSTRSKLSKDPKEKRDHMVLLEFVTAAGIKHGRDERYKTLPETGENLYFQSGGSHHHHHH	30416	10.32	+35
**NICD**				
Before SUMO hydrolysis:<br><br>HisSUMO-NICD-AviTag-Strep	MVHHHHHHGSDSEVNQEAKPEVKPEVKPETHINLKVSDGSSEIFFKIKKTTPLRRLMEAFAKRQGKEMDSLRFLYDGIRIQADQTPEDLDMEDNDIIEAHREQIGGSGGSNASCVGGVLWQRRLRRLAEGISEKTEAGSEEDRVRNEYEESQWTGERDTQSSTVSTTEAEPYYRSLRDFSPQLPPTQEEVSYSRGFTGEDEDMAFPGHLYDEVERTYPPSGAWGPLYDEVQMGPWDLHWPEDTYQDPRGIYDQVAGDLDTLEPDSLPFELRGHLVGGSGGSGLNDIFEAQKIEWHEGGSLPETGGGSWSHPQFEK	35575	4.68	-31
After SUMO hydrolysis:<br><br>NICD-AviTag-Strep	SGGSNASCVGGVLWQRRLRRLAEGISEKTEAGSEEDRVRNEYEESQWTGERDTQSSTVSTTEAEPYYRSLRDFSPQLPPTQEEVSYSRGFTGEDEDMAFPGHLYDEVERTYPPSGAWGPLYDEVQMGPWDLHWPEDTYQDPRGIYDQVAGDLDTLEPDSLPFELRGHLVGGSGGSGLNDIFEAQKIEWHEGGSLPETGGGSWSHPQFEK	23352	4.33	-25

4. SUPPLEMENTARY REFERENCES

(1) Scott, D. J.; Gunn, N. J.; Yong, K. J.; Wimmer, V. C.; Veldhuis, N. A.; Challis, L. M.; Haidar, M.; Petrou, S.; Bathgate, R. A. D.; Griffin, M. D. W. A Novel Ultra-Stable, Monomeric Green Fluorescent Protein For Direct Volumetric Imaging of Whole Organs Using CLARITY. *Sci Rep* **2018**, *8* (1), 1–15. https://doi.org/10.1038/s41598-017-18045-y.

(2) Lawrence, M. S.; Phillips, K. J.; Liu, D. R. Supercharging Proteins Can Impart Unusual Resilience. *J Am Chem Soc* **2007**, *129* (33), 10110–10112. https://doi.org/10.1021/ja071641y.

(3) McNaughton, B. R.; Cronican, J. J.; Thompson, D. B.; Liu, D. R. Mammalian Cell Penetration, SiRNA Transfection, and DNA Transfection by Supercharged Proteins. *Proc Natl Acad Sci U S A* **2009**, *106* (15), 6111–6116. https://doi.org/10.1073/pnas.0807883106.

(4) Altenburg, W. J.; Yewdall, N. A.; Vervoort, D. F. M.; van Stevendaal, M. H. M. E.; Mason, A. F.; van Hest, J. C. M. Programmed Spatial Organization of Biomacromolecules into Discrete, Coacervate-Based Protocells. *Nat Commun* **2020**, *11* (1), 1–10. https://doi.org/10.1038/s41467-020-20124-0.

(5) Zhang, M.; Chang, H.; Zhang, Y.; Yu, J.; Wu, L.; Ji, W.; Chen, J.; Liu, B.; Lu, J.; Liu, Y.; Zhang, J.; Xu, P.; Xu, T. Rational Design of True Monomeric and Bright Photoactivatable Fluorescent Proteins. *Nat Methods* **2012**, *9* (7), 727–729. https://doi.org/10.1038/nmeth.2021.

(6) Poudyal, R. R.; Guth-Metzler, R. M.; Veenis, A. J.; Frankel, E. A.; Keating, C. D.; Bevilacqua, P. C. Template-Directed RNA Polymerization and Enhanced Ribozyme Catalysis inside Membraneless Compartments Formed by Coacervates. *Nat Commun* **2019**, *10* (1), 490. https://doi.org/10.1038/S41467-019-08353-4.
